# Preferences of women for maternal healthcare services in low-income and middle-income countries: a systematic review of discrete choice experiments

**DOI:** 10.1136/bmjgh-2024-017410

**Published:** 2025-08-07

**Authors:** Daniel Gashaneh Belay, Gizachew A. Tessema, Melaku Birhanu Alemu, Bereket Kefale, Jennifer Dunne, Richard Norman

**Affiliations:** 1 Curtin School of Population Health, Curtin University, Perth, Western Australia, Australia; 2 Department of Epidemiology and Biostatistics, Institute of Public Health, College of Medicine and Health Sciences, University of Gondar, Gondar, Ethiopia; 3 School of Public Health, University of Adelaide, Adelaide, South Australia, Australia; 4 Department of Health Systems and Policy,Institute of Public Health, College of Medicine and Health Sciences, University of Gondar, Gondar, Ethiopia; 5 Department of Reproductive and Family Health, School of Public Health, College of Medicine and Health Sciences, Wollo University, Dessie, Ethiopia; 6 Dementia Centre of Excellence, enAble Institute, Curtin University, Perth, Western Australia, Australia

**Keywords:** Systematic review, Global Health, Health services research, Maternal health, Health policies and all other topics

## Abstract

**Background:**

Maternal healthcare service utilisation during pregnancy, childbirth and the subsequent postpartum periods could improve the health outcomes of women and newborns. However, women in low-income and middle-income countries (LMICs) have a lower uptake of these services, which is partly attributed to a lack of access to preferred maternal health services. This study systematically synthesised evidence on women’s preferences for maternal healthcare services in LMICs.

**Methods:**

A systematic search was undertaken from PubMed/MEDLINE, EMBASE, PsycINFO, CINAHL, Scopus and Global Health databases, and supplemented with Google Scholar for grey literature. We have included published articles from 1 January 2000 to the date of the last search, 14 July 2023. Studies were included if they reported preferences of women for maternal health services in LMICs using the stated preference analysis methods. The quality of the included papers was assessed using the conjoint analysis applications in the healthcare checklist. We have thematically presented the attributes using a healthcare access framework (accessibility, availability, accommodation, affordability and acceptability). The first two most important and least important attributes in each study were identified based on the relative importance scores.

**Findings:**

Of the 54 articles identified for full-text review, 15 studies from eight LMICs met the inclusion criteria for the final review. Attributes related to the acceptability of healthcare services, such as a provider’s good attitude and rapport (47.1%), and the availability of services, such as medications and supplies (41.2%), were typically considered the most important by women. Conversely, accessibility attributes, such as increased distance or travel time to health facilities (29.4%), and affordability attributes, such as increased cost of services (23.5%), were generally less valued by women.

**Interpretations:**

The acceptability and availability attributes of healthcare services were considered the most important by women. Aligning maternal healthcare service provision with women’s preferences can promote person-centred care, leading to increased service uptake.

**PROSPERO registration number:**

CRD42023444415.

**WHAT IS ALREADY KNOWN ON THIS TOPIC**

- Most maternal mortalities related to pregnancy and childbirth complications occur in low-income and lower-middle-income countries. Comprehensive maternal health services can significantly reduce these deaths; however, in addition to their limited availability, their uptake by women remains low in resource-limited countries. Both the demand-side and supply-side barriers prevent women from receiving or seeking care during pregnancy and childbirth in low-income and middle-income countries (LMICs).

**WHAT THIS STUDY ADDS**

- We have identified the most important and least important attributes influencing maternal health service uptake in LMICs. Our findings support attributes related to the acceptability of healthcare services such as the provider’s attitude and rapport, and availability of services such as medications and supplies were typically considered the most important by women. Conversely, accessibility attributes such as increased distance or travel time to health facilities, and affordability attributes, such as increased cost of services, were generally valued less by women.

**HOW THIS STUDY MIGHT AFFECT RESEARCH, PRACTICE OR POLICY**

- This evidence indicates a shift in priorities in maternal healthcare, challenging the conventional focus on geographical and financial barriers as primary concerns. Policies should prioritise investments in supply chain management to ensure the consistent availability of medications and essential supplies. Additionally, compassionate and respectful care should be integrated into health sector plans in LMICs. Findings from our review highlight the need for further mixed-methods research to gain deeper insights into the reasons behind women’s preferences.

## Introduction

 Almost 95% of global maternal mortality related to pregnancy and childbirth complications occurs in low-income and lower-middle-income countries.^1 2^ This can be reduced significantly through the provision of comprehensive maternal health services such as antenatal care (ANC), facility delivery and postnatal care (PNC).^3^ However, there is a low uptake rate of these services, especially in resource-limited countries.^2^ The WHO report shows that only 68% of all births in low-income countries and 78% in lower-middle-income countries were assisted by skilled health personnel compared with 99% in high-income and upper-middle-income countries in 2020.^2^

Different factors prevent women from seeking care during pregnancy and childbirth. These barriers to accessing health services can stem from both the demand and the supply sides.^4^ Supply-side determinants are aspects inherent to the health system that hinder service uptake by individuals, households or communities. The availability, accessibility and/or quality of maternal health service are the main supply determinants which often result in low utilisation of services.^4 5^ Health facilities in lower-income and low-income countries may lack the required expertise and facilities in terms of availing competent staff and equipment.^5^ For example, lack of medications and supplies in the health service is a factor which hinders women from utilisation of maternal healthcare uptake.^6^

While our review primarily focuses on identifying women’s preference for supply-side determinants of healthcare access barriers, it is also essential to consider demand-side determinants. Demand-side determinants are factors influencing the ability or willingness of individuals, households or communities to access health services at the same level.^4^ These are consumer-related factors such as low-income status, lack of access to education, affiliation with certain religions and gender norms which may hinder women from accessing healthcare services.^2^ In low-income and middle-income countries (LMICs), although maternal healthcare services are mostly provided free of charge, the expenses related to transportation to reach the healthcare facility, the income lost during the visit and the potential need to pay for medication can hinder access to these services.^7^

The interaction between supply-side and demand-side determinants is crucial in understanding maternal healthcare utilisation in LMICs. Research indicates that even when healthcare services are available and providers are well-trained, low demand due to cultural norms, lack of health literacy or financial constraints may still limit service utilisation.^8^ Conversely, strong demand for maternal healthcare can be hindered by inadequate supply, such as stockouts of essential medications or negative provider attitudes.^9^ Understanding these dynamics is crucial for designing holistic interventions that address both structural barriers and individual-level determinants to improve maternal health outcomes in LMICs.^10^

To increase the low uptake of maternal healthcare services and prevent maternal morbidity and mortality, it is important to focus on attributes that are preferred and perceived by women as a determinant of maternal health service utilisation.^11 12^ Thus, the lives of women in LMICs could be saved by implementing reproductive and maternal health interventions which involve women themselves as decision-makers.^13^ To elicit consumer (ie, women) preferences and shared decision-making, stated preference methods such as conjoint analysis are commonly used in research.^14^ Conjoint analysis is a statistical technique used to understand how people make complex choices.^15^ It breaks down a product, service or intervention into its constituent attributes and examines how individuals prioritise these attributes when deciding.^16^ In the context of evaluating stated preferences in maternal health services, conjoint analysis is useful for assessing which aspects of healthcare services (such as cost, distance to the facility, quality of care and provider attitude) are most valued by women when making decisions about maternal healthcare.^16^ Since it prioritises service users’ preferences and these preferences reflect individual or social welfare, stated preference techniques help formulate policies to enhance social welfare, including improving service coverage.^17^

In recent years, there has been a growing interest in and extensive adoption of preference elicitation methods in many low-income countries.^12^ However, there is limited evidence evaluating women’s preference for maternal healthcare in resource-limited countries.^18^ While there was one systematic review focusing on reproductive and maternal health services in sub-Saharan Africa,^12^ this included a limited number of studies, most were focused on reproductive services.^12^ Addressing this gap is crucial because understanding women’s preferences is fundamental to improving maternal health outcomes in resource-limited settings. Therefore, this study aims to synthesise available evidence on women’s preferences for maternal healthcare services in LMICs. By shedding light on these preferences, this research will contribute by identifying the most important attributes to informing policies and interventions that better meet the needs of women in such settings. Identifying these specific needs and preferences will enable policymakers and healthcare providers to design interventions tailored to improve maternal healthcare effectively.

## Methods

A systematic review of published studies reporting stated preferences for maternal healthcare services (ANC, childbirth services and PNC) in LMICs was conducted. The review protocol was prospectively registered in the International Prospective Registration of Systematic Reviews (PROSPERO) and publicly available (registration # CRD42023444415).

### Data sources and search strategy

Electronic databases, including MEDLINE, EMBASE, PsycINFO, Scopus, Global Health and CINAHL, were used to search for published articles. The search terms included the three main search concepts, which were “preference”, “maternal health services” and “low- and middle-income countries”. Synonyms for these words were searched, and a keywords and medical heading search was also conducted (online supplemental table 1). The list of LMICs was taken from the World Bank 2022 report.^19^ Additionally, Google Scholar was used to identify relevant grey literature. Then, the first 200 results returned were taken. Additionally, to capture articles that may have been mis-indexed, a further search was conducted using the reference lists of the included studies. The Preferred Reporting Items for Systematic Reviews and Meta-Analyses flow chart and guidelines were used to summarise the screening process of this systematic review.^20^ The databases were searched on 13 and 14 July 2023.

### Eligibility criteria and study selection

Studies were eligible for inclusion if they fulfilled the following criteria: (1) study population: women of reproductive age; (2) outcome: stated preference for maternal health services (ANC, child delivery care and PNC); (3) study design: observational study design with conjoint analysis such as discrete choice experiment (DCE), Thurstone and best worst which are a quantitative estimation of preferences for a product, service or intervention using its attributes; (4) effect estimate: studies reporting measure of preferences (eg, beta-coefficient, OR) with 95% CIs; and (5) setting: LMICs based on the World Bank 2022 report^19^ (online supplemental table 2). Studies were excluded if they were qualitative studies, case studies, case series, systematic reviews, conference presentations and commentaries (online supplemental table 3).

### Screening process

The records identified from the electronic databases were exported to Endnote V.9 software to manage and remove duplicates. The remaining records were transferred to Rayyan^21^ and screened by title and abstract, independently by two reviewers (DGB and BK). The same reviewers (DGB and BK) performed the full-text review of the identified articles. Both reviewers made decisions regarding the studies’ inclusion/exclusion, and the primary reason for exclusion was documented. Other reviewers (RN, GT and JD) resolved the disagreements between the reviewers during the review process.

### Data extraction and data analysis

Relevant data such as authorship, country, publication year, population, sampling methods, attribute and level identification, the analysis method used, type of maternity service and preferences of study participants were extracted (online supplemental table 4). The principal investigator (DGB) extracted the data independently from the included studies, and another reviewer (MBA) reviewed the extraction.

A descriptive analysis was used to summarise the studies. The importance of an attribute was evaluated by comparing the relative importance scores of all attributes within a study; the attribute with the highest relative importance score was considered the most important attribute.

Based on this, the first two most important attributes and the least important attribute in each study were identified. The included studies were further thematised and synthesised according to identified attributes.^22^ For the thematic synthesis of the included study, we used the dimensions of the healthcare access framework, which are accessibility, availability, accommodation, affordability and acceptability.^4 22 23^ ‘Availability’ is defined as the volume and type of healthcare services meeting patients’ demands. ‘Accessibility’ is the physical access to healthcare services, depending on the geographical distribution of healthcare facilities, patients’ residential locations, transportation resources and travel time. Accommodation can be defined as the organisation of healthcare services to fulfil patients’ needs, the client’s ability to accommodate these factors and the client’s perception of their appropriateness. Affordability is the pricing of healthcare services in relation to patients’ ability to pay. Acceptability refers to patients’ attitudes towards healthcare services and providers.^4 22 23^ The dimensions of access are interrelated and influence each other.^22^ This framework is used to identify interventions that effectively address specific healthcare access barriers, particularly in underserved populations. Unlike other frameworks (eg, Andersen’s Behavioral Model), which emphasise individual determinants of healthcare use,^24^ the Healthcare Access Framework focuses on systemic (supply-side) factors affecting healthcare access.

The attributes from the included studies were coded line-by-line. Codes were then grouped under one of the five healthcare access dimensions they most closely aligned with. Themes were identified within each dimension based on the relative importance of included attributes. These themes provided a deeper understanding of how each dimension influenced women’s access to maternal healthcare services (online supplemental tables 5 and 6).

### Assessment of the quality of the individual studies

The conjoint analysis applications in the healthcare checklist by the International Society for Pharmacoeconomics and Outcomes Research (ISPOR)^25^ were adopted to assess the quality of the included studies. This is a 10-item checklist that includes items relating to the (1) research question; (2) attributes and levels; (3) construction of tasks; (4) experimental design; (5) preference elicitation; (6) instrument design; (7) data collection plan; (8) statistical analyses; (9) results and conclusions; and (10) study presentation. Each 10 item has 3 subitems, and the score is calculated based on the sum of these 30 subitems (with a maximum total of 30 points). The subitems were rated as ‘Yes’ or ‘No’ depending on whether the study described or mentioned each subitem. If the study included or mentioned a subitem, it received a ‘Yes’ score; otherwise, it received a ‘No’ score. Based on this, we classified 25–30 points as ‘good’, 20–25 points as ‘moderate’ and <20 points as a ‘low’ score. Two reviewers (BK and DGB) independently assessed the risk of bias in each study, and the discrepancies in the score given resolved through justification in a round-table discussion. Cohen’s kappa result (0.438) indicates that there was a moderate agreement between the two reviewers (online supplemental table 7).

## Results

**Figure 1:**
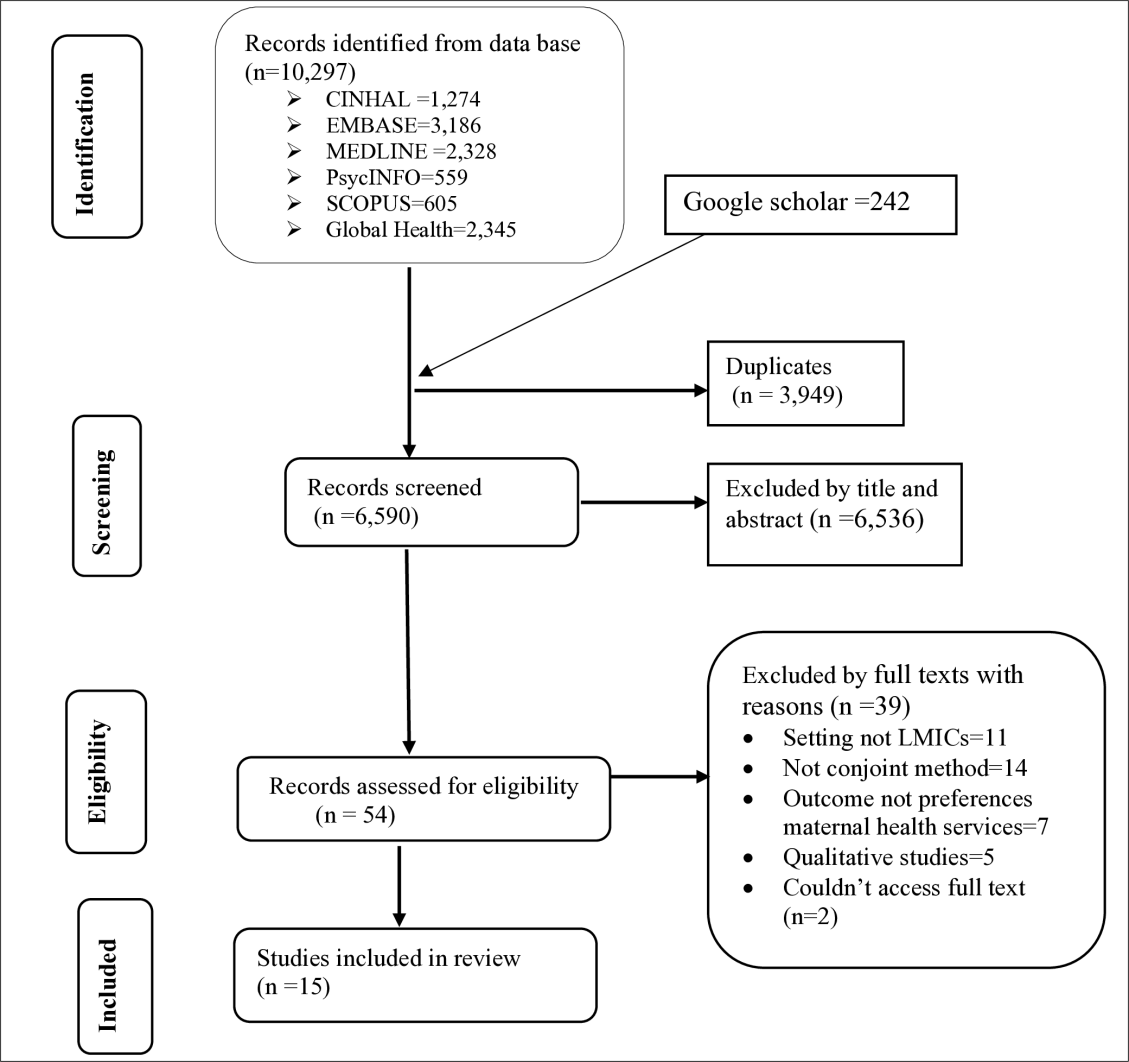
Preferred Reporting Items for Systematic Reviews and Meta-Analyses diagram for the study selection strategies of women’s preferences for maternal healthcare in low-income and middle-income countries (LMICs).

From the 10 485 records initially identified, 3949 were excluded as duplicates. A further 6536 records were excluded during the title and abstract screening, leaving 54 studies for full-text assessment (figure 1). Out of 54 articles assessed for eligibility, 39 articles were excluded for reasons such as not being from LMICs (n=11), the method not being conjoint analysis (n=14), the outcome not being preferences of maternal health services (n=7) and qualitative studies (n=5) (online supplemental table 3). Despite our attempts to contact the corresponding authors by email, we were unable to access the full texts of two studies,^26 27^ which were included during screening. Finally, a total of 15 studies were included in the final review (figure 1).

### Study and sample characteristics

From the total of 15 articles included, there were 9987 participants (with a range of 136–3238 respondents). The participant’s response rate ranged from 73.3% to 100%. The age of the participants ranged from 15 to 55 years, with a mean age of 17.2–35.8 years. The studies were conducted in Ethiopia,^18 28 29^ Kenya,^6 30 31^ Tanzania,^32^,^35^ Nigeria,^36^ Bangladesh,^11^ India,^37^ China^38^ and Argentina,^39^ and were conducted from 2007 to 2023, with two-thirds (n=10) of the studies conducted after 2015 (table 1).

**Table 1:** Study and sample characteristics (n=15)

Characteristics	Level	N (%)
Country	Argentina	1 (6.7)
	Bangladesh	1 (6.7)
	China	1 (6.7)
	Ethiopia	3 (20)
	India	1 (6.7)
	Kenya	3 (20)
	Nigeria	1 (6.7)
	Tanzania	4 (26.6)
Publication year	<2015	5 (33.3)
	>2015	10 (66.7)
Study setting	Community	8 (53.3)
	Health facility	6 (40)
	Both	1 (6.7)
Sample size	<500	10 (66.7)
	500–1000	2 (13.3)
	>1000	3 (20)
Mean age	<24	3 (20)
	>24	12 (80)
Response rate	<80%	1 (6.7)
	>80%	14 (93.3)
Methods used to identify attributes and attribute levels	Literature review	14 (93.3)
	Expert consultation	6 (40)
	Focus group discussion	8 (53.3)
	Interviews	12 (80)
	Pilot test	10 (66.7)
	Others (observation)	2 (13.3)
Maternal health service preferences	Antenatal care	3 (20)
	Health facility delivery care	12 (80)
Women’s current pregnancy status	Pregnant	5 (33.3)
	Delivery in the past 2 years	6 (40)
	Delivery in the past 5 years	3 (20)
	Pregnant or delivered in the past 2 years	1 (6.7)

### Quality assessment

All the included articles, except one, had good qualities (scored 25–30). All studies had a well-defined research question, attributes and levels of selection supported by evidence at least with literature reviews. More than half of the included articles (n=8)^618 28 31^,^34 40^ reported information on each item of the ISPOR checklist, except including other qualifying questions such as the strength and confidence of preference in addition to preference elicitation. Some studies strengthen the preference elicitation by doing qualitative analysis.^6 11 31 36^ One study has moderate qualities in which the properties of the experimental design were not evaluated and the quality of the responses was not examined^39^ (online supplemental table 7).

### Preferences of maternal health services and women’s current health condition

Of the 15 included studies, 5 studies^3237^,^40^ included currently pregnant women, while 6 studies^6 11 18 29 34 36^ included women who had given birth within the past 2 years (table 1).

The majority (80%) of the included studies assessed women’s perceived preferences for maternal healthcare services for childbirth,^611 18 28 29 31 33^,^35 37 39^ while the remaining three studies^32 38 40^ assessed the preferences of women for ANC services. Of those women who asked about childbirth service preference, most (50%) of them were women who gave birth in the past 5 years. From studies conducted on ANC service preferences, one study focused on depression treatment and perinatal service preferences^40^ while another study aimed at women’s preference for prenatal testing in maternal healthcare units for detecting genetic abnormalities.^38^ Of the total included, two studies had a subgroup analysis.^29 39^ The first study, conducted in Ethiopia, explored the preferences for obstetric care among women with specific patient groups such as depressive symptoms and post-traumatic stress disorder (PTSD).^29^ The second study, conducted in Argentina, was concerned with women’s preferences in modes of delivery in public and private hospitals^39^ (online supplemental table 6).

### Methods used to identify attributes and attribute levels

Different methods were used to determine the attributes and attribute levels for the surveys. Almost all (n=14) of the studies used a literature review as an attribute development framework. Qualitative study methods such as unstructured interviews (n=12),^611 18 28 29 31^,^33 36 37 39 40^ focus group discussion (n=8),^6 11 29 31 32 34 40^ and expert consultation (n=6)^1133 36^,^38 40^ were also the most commonly used methods (table 1). Most studies (n=11) conducted a pilot on the final list of attributes and attribute levels. However, only one of the studies^39^ provided details about the pilot, including the number of women included and a description of the pilot results.

### Experimental design

All the included studies were DCEs, and the experimental designs for the DCE questions were created in Sawtooth (n=7),^11 18 29 33 34 38^ Ngene (n=3),^28 37 40^ JMP (n=2)^6 31^ or SPSS (n=1)^39^ software. A total of 10 studies reported the total number of possible choice scenarios, which ranged from 96 to 600.^618 31^,^38^ Blocking was employed in five studies, with three of them using two blocks,^6 31 32^ one using three blocks^37^ and the remaining one using five blocks.^33^ The number of tasks per respondent ranges from 8 to 16, with an average of 10 choice tasks delivered to each respondent across all included studies. From all the included articles, except for three studies which have three choice options,^11 35 36^ all the other included articles have two choice options other than opt-out. Of these three studies, two^35 36^ did not include the opt-out choice option. Most of the included studies used a mixed logit model^6 31 32 34 40^ and STATA^28 32 34 39^ software to analyse women’s preferences for maternal health services (online supplemental table 8).

### Standard scripts and pictures

To ensure comprehension understanding among this low-literacy population, most studies used pictures, which can depict the choice tasks, including attributes and levels^1128 31^,^35^ (online supplemental table 4). To maintain consistency, most studies reported using standard scripts to inquire about the preferences of women for maternal health service utilisation (online supplemental table 9)

### Attribute classification

The studies included 91 attributes. The number of attributes included for the analysis within the studies ranged from 5 to 9, whereas the levels of attributes ranged from 2 to 5 (online supplemental table 4).

**Figure 2:**
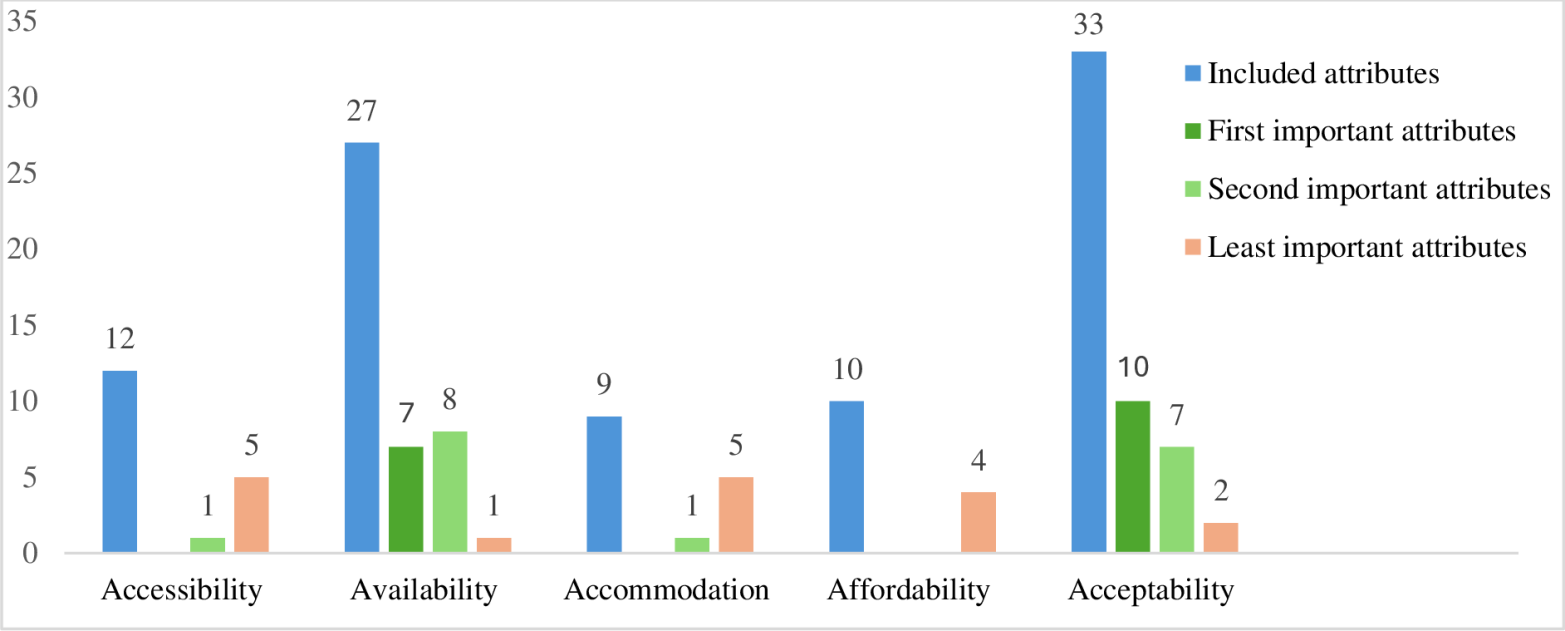
The included attributes and their relative importance.

Based on the dimensions access attribute classification framework, most studies included ‘acceptability’ and ‘availability’ dimensions of attributes within the analysis. Of the 91 included attributes in the studies, 33 (36.3%) were classified as acceptability attributes and 27 (39.7%) as availability attributes. The remaining attributes are classified as follows: 12 (13.2%) as accessibility attributes, 10 (11%) as affordability attributes and 9 (9.9%) as accommodation attributes (figure 2).

Healthcare provider characteristics such as attitude and rapport (n=12)^611 18 28 29 31^,^37^ were the most included attributes in the ‘acceptability’ dimension of care. Except for three studies,^38^,^40^ all studies included this attribute. Healthcare service cleanliness (n=6)^6 11 31 32 37 41^ was also the most included attribute in this dimension. The most included attributes in the ‘availability’ dimension of care were availabilities of medical equipment and drugs (n=9),^611 18 28 29 31 33^,^35^ and types of healthcare providers (n=9).^11 18 28 29 33 35 37^ Distance or travel time to health facility (n=7)^618 28 31^,^33 35^ and cost of healthcare service (n=10)^611 18 28 29 31 33^,^35 38^ were other commonly included attributes from ‘accessibility’ and ‘affordability’ dimensions, respectively. ‘Accommodation’ was the least included dimension, with waiting time (n=3)^11 37 38^ being the most included attribute (online supplemental table 6).

### Relative importance of attributes

Comparing the relative importance scores of all attributes within a study, the ‘acceptability’ dimension was selected as the first-ranked attribute in ten (58.8%) studies^629 31^,^33 36 39 41^ and the second-ranked attribute in seven (41.2%) studies.^1835^,^40^ For example, healthcare providers’ attitudes such as being respectful, kind, supportive, smiling and listening carefully were the most important ‘acceptability’ attributes reported by eight studies.^618 28 29 32^,^34 40^ Healthcare facilities’ cleanliness was also an important ‘acceptability’ attribute reported by two studies.^31 37^ The ‘availability’ dimension was considered the most important attribute in seven (41.2%) studies^11 18 28 35 37 38 40^ and the second most important attribute in eight (47.1%) studies.^6 11 29 31 33 41^ For example, the availability of medications and supplies was the most important (n=3)^18 28 35^ and the second most important (n=4)^6 11 31 33^ attributes for women choosing facilities for maternal healthcare in studies of five different LMICs. The availability of a continuum of maternal healthcare^11^ and ward visits by doctors^29^ or specialists^37^ were other important ‘availability’ attributes.

Most of the least important attributes were related to ‘accessibility’ dimension attributes such as increased distance^28 29^ and travel time to health facilities.^18 33^ Moreover, ‘accommodation’ dimension attributes such as increased waiting time,^37 38^ having the possibility of scheduling childbirth^39^ and low information delivery method^40^ and ‘affordability’ dimension attributes such as the increased cost of service^6 11 31 34^ were also the least important attributes (table 2 and online supplemental table 6).

**Table 2:** The relative importance of attributes for women’s stated preferences for maternal healthcare services in low-income and middle-income countries (n=17); (two studies had subgroup analysis)

Attributes (dimensions of access)	First important (%)	Second important (%)	Least important (%)
*Acceptability*	10 (58.8)	7 (41.2)	2 (11.8)
1. Provider attitudes	6 (35.3)	2 (11.8)	0 (0.0)
2. Sexual function after delivery	2 (11.8)	0 (0.0)	0 (0.0)
3. Healthcare facility cleanliness	1 (5.9)	1 (5.9)	1 (5.9)
4. Health system conditions	1 (5.9)	0 (0.0)	0 (0.0)
5. Quality of clinical services	0 (0.0)	0 (0.0)	1 (5.9)
6. Recovery after delivery	0 (0.0)	1 (5.9)	0 (0.0)
7. Pain during delivery	0 (0.0)	1 (5.9)	0 (0.0)
8. Sexual abuse	0 (0.0)	1 (5.9)	0 (0.0)
9. Detection rate	0 (0.0)	1 (5.9)	0 (0.0)
*Availability*	7 (41.2)	8 (47.1)	1 (5.9)
10. Availability of medications and supplies	4 (23.5)	3 (17.6)	0 (0.0)
11. Test procedure type	1 (5.9)	0 (0.0)	0 (0.0)
12. Types of healthcare providers	1 (5.9)	3 (17.6)	1 (5.9)
13. Content of care/procedure/service	1 (5.9)	1 (5.9)	0 (0.0)
14. Doctors’ medical knowledge	0 (0.0)	1 (5.9)	0 (0.0)
*Accessibility*	0 (0.0)	1 (5.9)	5 (29.4)
15. Distance from a health facility	0 (0.0)	1 (5.9)	5 (29.4)
*Affordability*	0 (0.0)	0 (0.0)	4 (23.5)
16. Cost of service	0 (0.0)	0 (0.0)	4 (23.5)
*Accommodation*	0 (0.0)	1 (5.9)	5 (29.4)
17. Support persons allowed	0 (0.0)	1 (5.9)	0 (0.0)
18. Waiting time	0 (0.0)	0 (0.0)	2 (11.8)
19. Possibility of scheduling	0 (0.0)	0 (0.0)	2 (11.8)
20. Types of information delivery systems	0 (0.0)	0 (0.0)	1 (5.9)

Moreover, the geographical distribution of stated preferences among women for maternal health services showed that women residing in countries such as Tanzania, Ethiopia, Kenya and Nigeria preferred attributes related to the acceptability of health services, while women living in India, China and Bangladesh prioritised the availability of health services. Women who live in China and Argentina give less value to accommodation attributes, while in Ethiopia, accessibility attributes are given less priority, and in India, Bangladesh and Kenya, affordability attributes are given less priority (online supplemental figure 1).

### Preference interactions

Nearly half of the included studies (n=9)^611 28 29 31 32 34^,^36^ assessed the effect of participants’ sociodemographic and/or disease-specific characteristics on their maternal health service preferences (online supplemental table 10). Women of high education status (ie, secondary or above education) preferred maternity care services which have technical qualities such as having quality clinical care,^6 31^ doctors with ‘excellent’ medical knowledge^34^ and availability of equipment and drugs.^35^ Moreover, non-technical qualities of maternal healthcare, such as no mistreatment related to sexual abuse,^36^ maintaining privacy^34^ and kind and friendly staff,^32^ were also more important attributes to women with high education status.

Women who had better income status in their household had a preference for the availability of referral services at a health facility,^6^ clean health facilities^31^ and short walking distance.^35^ Moreover, women from urban areas showed a preference for clean facilities, availability of a shaded waiting area^32^ and facilities with kind and helpful staff.^11 32^ While rural women emphasised short waiting times as a relatively important attribute.^11^

Primiparous women showed a higher preference for privacy,^34^ for the clinic to be clean^32 34^ and for the availability of seating^32^ than women who had delivered more children.

### Validity and reliabilities

Some studies compared the sociodemographic characteristics of the study population with those of the demographic and health survey, to assess the extent to which the sample characteristics were matched, aiding in the evaluation of the external validity of the study outputs.^18 28^ Moreover, interviewer and respondent genders were matched to reduce socially desirable answers and response bias.^28^ Some other studies also checked the theoretical validity by comparing the expected direction of the parameter estimates.^6 31 37^

Studies were used to compare the modelled preferences with the preference of holdout tasks (fixed-choice card) to test the predictive validity of the utility estimates for facility choices.^18 33 34^ These are choice cards in which the attribute-level combinations in the tasks remain fixed (held constant) across respondents.^37 42^ They serve as a validation tool to assess the consistency and predictive accuracy of respondents’ choices. Moreover, the reliability of the choices (choice consistency) was measured by comparing respondent choices in the repeat choice set and the original choice set and counting the number of times the same alternative was chosen.^34^

## Discussion

Identifying the attributes that influence the preferences of women for maternal health services can provide policymakers and health professionals with information to improve the health system’s responsiveness to women’s needs and expectations in LMICs.^43^ This review showed the most important attributes were attributes related to the ‘acceptability’ dimension, including provider attitudes such as being respectful, kind, supportive, having good rapport, smiling and listening carefully. Moreover, the ‘availability’ dimensions of healthcare services such as the availability of medical equipment and drugs were measured as the most important attributes. While the ‘affordability’ attributes such as increased cost of services and ‘accessibility’ attributes such as increased distance or travel time to health facilities were given less value by women. Moreover, there are geographical differences in women’s preferences for maternal health service attributes in LMICs.

This review showed that women valued more on relational quality of maternal healthcare such as a respectful and kind healthcare worker above the inputs of the healthcare facility. This is supported by a systematic review study in Sub-Saharan Africa.^44^ A friendly staff attitude, which means avoiding rude staff, is the second most important attribute for preferences study regarding hospital quality in Zambia.^45^ Women’s preferences for place of delivery and willingness to deliver in a health facility were strongly and negatively associated with disrespect and abuse during previous institutional birth.^46^

The WHO quality of care standard states that women should be treated respectfully and in a way that upholds their dignity.^47^ In some LMICs, compassionate and respectful care is prioritised in health sector plans.^3 48^ However, evidence has shown that the mistreatment of women during labour and delivery presents a significant challenge in different LMICs such as Ethiopia,^49^ Nigeria^50^ and Tanzania^51^ which has been increasingly recognised as a major barrier to women accessing health facilities.^6^ This underscores the need for policies and practices that prioritise respectful and compassionate care. Addressing these issues is crucial for improving maternal health outcomes and ensuring that women feel valued and dignified during their healthcare experiences.

In this study, the ‘availability’ of healthcare services, such as the availability of medical equipment and drugs,^6 11 18 28 31 33 36^ and the individual choice of healthcare provider (doctors or specialists)^29 37^ were measured as the most important attributes. A systematic review comparing the relative importance of structure, process and outcome attributes of women’s preferences for reproductive and maternal healthcare indicated that the structure of the healthcare service was more important than the process and outcome attributes.^12^ The study in Zambia^45^ also showed that technical quality of care represented by the thoroughness of examination was the most important attribute for the preferences for quality hospitals. Similarly, a study in Tanzania^52^ showed that the odds of household healthcare utilisation were three times higher with health facilities which had continuous availability of all essential medicines. WHO also states that access to treatment is heavily dependent on the availability of affordable medicines.^53^ On the other hand, inadequate supplies were identified as the major barrier to the care process, such as providing low levels of compassionate and respectful care.^54^ Therefore, to improve the uptake of maternal health services, policies should focus on enhancing the input attributes of healthcare facilities that matter to women in this study. This includes providing a continuum of maternal healthcare with senior healthcare providers and ensuring the continuous availability of essential medicines.

However, in our review, attributes such as ‘affordability’ (increased cost of services),^6 11 31 34^ and ‘accessibility’ (increased travel time or distance from health facilities) were found to be the least important. The reason why the cost of services is a less important attribute in this review is that, in most developing countries, delivery services were made free at public health facilities to improve maternal health outcomes.^55 56^ The free delivery policy substantially reduced out-of-pocket costs^31^; as a result, women gave less value. The lower value assigned to distance in our study contrasts with previous reviews that reported distance as one of the predominant barriers to accessing maternal healthcare.^57^ This might show that other attributes that signal quality such as positive attitudes of healthcare workers and the availability of drugs were the most important than short-distance travel.^31 33^ Moreover, it may also be explained that women could get geographical access to several healthcare facilities^31^ through public ambulance services.

In this review, women of high education status preferred maternity care which has technical^6 31^ and non-technical qualities.^32^ Cross-country analyses have also found that an individual’s level of education affects her rating of health system responsiveness on standardised vignettes.^58^ Since educated women are more likely to have access to quality health services and greater exposure to media, their expectations also increase.^34 59^ This is because the preference for modern medical equipment and drugs increased with increased media exposure.^34^ Better-educated women tend to be more informed about healthcare and have the chance to choose it. Therefore, to improve professional maternal healthcare utilisation, there is a need to focus on women’s education beyond the primary level.^60^

Our review has also shown that women with higher wealth status tend to prioritise attributes such as the availability of referral services,^6^ clean health facilities^31^ and a short walking distance.^6 35^ Women with higher economic status are likely to have higher expectations for care and can afford referral services.^34^ Wealthy women mostly reside in urban settings where most services are within short walking distance.^61^ Moreover, the opportunity costs of walking to distant health facilities might be higher for wealthier women^35^ which leads to preferring services found in short distances.

This review also shows that women residing in sub-Saharan African countries such as Tanzania, Ethiopia, Kenya and Nigeria preferred attributes related to the acceptability of maternal health services. This might be due to cultural beliefs and traditions which strongly influence healthcare-seeking behaviour. Women may prioritise healthcare providers who respect their values, privacy and traditional birthing practices.^62^ Moreover, mistreatment, or lack of culturally competent care, may deter women from using services. Evidence shows that mistreatment of women during labour and delivery presents a significant challenge in countries such as Ethiopia,^49^ Nigeria^50^ and Tanzania.^51^

Moreover, women’s current health status may affect their maternal health preferences. A study conducted in Ethiopia among women with high depression or PTSD^29^ showed that healthcare provider’s positive attitudes, such as smiling and listening carefully, were considered disutility attributes for choosing maternal healthcare. This might be because women with psychopathology, such as high depressive symptoms or PTSD, had lower trust in providers, which in turn led to less concern about their interpersonal skills.^29^ The variation in women’s preferences for maternal health services across sociodemographic characteristics and other conditions may indicate the need for services to be tailored to reflect the local communities.

### Strengths and limitations

We have employed standard and rigorous approaches to summarise and present evidence on women’s stated preferences regarding maternal health services in LMICs. As we are solely concentrating on the three maternal health services (ANC, childbirth services and PNC), the attributes were relatively similar. However, since most studies, except three, focused on preferences for childbirth services, comparing the findings between these maternal health services was impossible. Furthermore, since the magnitude of preferences in choice experiments is often measured relative to other attributes, we cannot generate either pooled estimates for each attribute or compare the strength of the estimate between studies. Additionally, some dimensions of the healthcare access framework are interrelated, making it difficult to categorise attributes under them.

## Conclusion

Aligning maternal healthcare service provision with women’s preferences can promote person-centred care, leading to increased service uptake. Therefore, policies should focus on enhancing the key attributes of healthcare facilities that matter to women in this study, such as ensuring the continuous availability of essential medicines. Compassionate and respectful care should also be prioritised in the health sector plans of LMICs. Moreover, empowering women through education and income generation plays a significant role in positively shaping these preferences, necessitating targeted strategies to address diverse needs. Furthermore, implementing a mixed-methods approach that combines qualitative and quantitative methods to gain deeper insights into the reasons behind women’s preferences and longitudinal research to understand how preferences and priorities change over time, especially in response to policy changes or interventions, is needed.

## Supplementary material

10.1136/bmjgh-2024-017410 online supplemental file 1

## Data Availability

No data are available.
