# Supplementary material for: Preferences of women for maternal healthcare services in low-income and middle-income countries: a systematic review of discrete choice experiments
Source: BMJ Glob Health. 2025 Aug 7;10(8):e017410. doi: 10.1136/bmjgh-2024-017410 (PMC12336509; doi:10.1136/bmjgh-2024-017410)
Supplement: online supplemental file 1 [file bmjgh-10-8-s001.pdf]

List of Supplementary Tables

Supplementary Table 1: Search strategy for preferences of women for maternal healthcare services in LMICs

	**CONCEPT 1 – Preference**	**CONCEPT 2 – Maternal health services**	**CONCEPT 3 – low- and middle-income countries**
**Keywords (Ovid)**	((discrete or discrete-choice or stated or thurstone or conjoint or DCE or DCEs) ADJ3 (choice or experiment or valuation or method* or analysis or preference)). ti,ab. or<br>(preference* OR "stated-preference*" OR DCE or DCE* OR "best worst" OR BWM OR BWS OR conjoint* OR thurstone OR WTP OR "willingness to pay" OR "willingness to accept" OR WTA OR "patient weighting" OR "patient rating" OR "patient ranking" OR "patient perspective").ti,ab.	(delivery OR obstetric OR antenatal OR postnatal OR matern* OR pregnan*). ti,ab. OR ((ANC OR prenatal OR perinatal OR pri-birth) ADJ2 (utili?ation* OR service* OR care* OR visit* OR book* OR check* OR "check-up*" OR "follow up")). ti,ab. OR ((childbirth OR "child birth" OR "child bear*")). ti,ab. OR ((PNC OR postpartum OR "postpartum") ADJ2 (services* OR care* OR visit* OR book* OR check* OR "ch eck-up*" OR "follow up")). ti,ab.	((Developing or undeveloped or "low income" or "low-income*" or "middle income" or "middle-income" or "low resource" or "middle resource" or "low-and middle- income" or "low and middle income" or "non-industrialised" or "third world") ADJ2 (countr* or nation* or setting*)).ti,ab. OR (Africa, "sub-Sahara" or "sub-Saharan" or "Eastern Africa" or "Middle East Asia" or Asia or "Latin America" or "South America").ti,ab. OR (Afghanistan or Burkina Faso or Burundi or Central African Republic or Chad or Congo Democratic Republic or Eritrea or Ethiopia or Gambia or Guinea or Guinea-Bissau or Korea Democratic People Republic or Liberia or Madagascar or Malawi or Mali or Mozambique or Niger or Rwanda or Sierra Leone or Somalia or South Sudan or Sudan or Syrian Arab Republic or Togo or Uganda or Yemen Republic or Zambia or Angola or Algeria or Bangladesh or Benin or Bhutan or Bolivia or Cabo Verde or Cambodia or Cameroon or Comoros or Congo Republic or Cote d Ivoire or Djibouti or Egypt Arab Republic or El Salvador or Eswatini or Ghana or Haiti or Honduras or India or Indonesia or Iran Islamic Republic or Kenya* or Kiribati or Kyrgyz Republic or Lao People Democratic Republic or Lebanon or Lesotho or Mauritania or Micronesia Fed Sts or Mongolia or Morocco or Myanmar or Nepal or Nicaragua or Nigeria or Pakistan or Papua New Guinea or Philippines or Samoa or "Sao Tome and

			principe" or Senegal or Solomon Islands or Sri Lanka or Tanzania or Tajikistan or Timor-Leste or Tunisia or Ukraine or Uzbekistan or Vanuatu or Vietnam or "West Bank and Gaza" or Zimbabwe or Albania or American samoa or Argentina or Armenia or Azerbaijan or Belarus or Belize or "Bosnia and Herzegovina" or Botswana or Brazil or Bulgaria or China or Colombia or Costa Rica or Cuba or Dominica or Dominican Republic or Equatorial Guinea or Ecuador or Fiji or Gabon or Georgia or Grenada or Guatemala or Guyana or Iraq or Jamaica or Jordan or Kazakhstan or Kosovo or Libya or Malaysia or Maldives or Marshall Islands or Mauritius or Mexico or Moldova or Montenegro or Namibia or North Macedonia or Palau or Paraguay or Peru or Russian Federation or Serbia or South Africa or Saint Lucia or "Saint Vincent and the Grenadines" or Suriname or Thailand or Tonga or Turkiye or Turkmenistan or Tuvalu).ti,ab.
**Keyword (EBSCO)**	((discrete or discrete-choice or stated or thurstone or conjoint or DCE or DCEs) N3 (choice or experiment or valuation or method* or analysis or preference)) or<br><br>(preference* OR "stated preference*" OR DCE or DCE* OR "best worst" OR BWM OR BWS OR conjoint* OR Thurstone OR WTP OR "willingness to pay" OR "willingness to accept" OR WTA OR "patient weighting" OR "patient rating"	(delivery OR obstetric OR antenatal OR postnatal OR matern* OR OR pregnan*) OR ((ANC OR prenatal OR perinatal OR pri-birth) N2 (utili?ation* OR service* OR care* OR visit* OR book* OR check* OR "check-up*" OR "follow up")) OR ((childbirth OR "child birth" OR "child bear*")) OR ((PNC OR postpartum OR "postpartum") N2 (services* OR care* OR visit*	((Developing or undeveloped or "low income or low-income"* or "middle income" or "middle-income" or "low resource" or "middle resource" or "low-and middle- income" or "low and middle income" or "non-industrialised" or "third world") N2 (countr* or nation* or setting*)) OR (Africa, sub-Sahara or sub-Saharan or Eastern Africa or Middle East Asia or Asia or Latin America or South America) OR (Afghanistan or Burkina Faso or Burundi or Central African Republic or Chad or Congo Democratic Republic or Eritrea or Ethiopia or Gambia or Guinea or Guinea-Bissau or Korea Democratic People Republic or Liberia or Madagascar or Malawi or Mali or Mozambique or Niger or Rwanda or Sierra Leone or Somalia or South Sudan or Sudan or Syrian Arab Republic or Togo or Uganda or Yemen Republic or Zambia or Angola or Algeria or Bangladesh or Benin or Bhutan or Bolivia or Cabo Verde

	OR "patient ranking" OR "patient perspective")	OR book* OR check* OR "check-up*" OR "follow up"))	or Cambodia or Cameroon or Comoros or Congo Republic or Cote d Ivoire or Djibouti or Egypt Arab Republic or El Salvador or Eswatini or Ghana or Haiti or Honduras or India or Indonesia or Iran Islamic Republic or Kenya or Kiribati or Kyrgyz Republic or Lao People Democratic Republic or Lebanon or Lesotho or Mauritania or Micronesia Fed Sts or Mongolia or Morocco or Myanmar or Nepal or Nicaragua or Nigeria or Pakistan or Papua New Guinea or Philippines or Samoa or "Sao Tome and principe" or Senegal or Solomon Islands or Sri Lanka or Tanzania or Tajikistan or Timor-Leste or Tunisia or Ukraine or Uzbekistan or Vanuatu or Vietnam or "West Bank and Gaza" or Zimbabwe or Albania or American samoa or Argentina or Armenia or Azerbaijan or Belarus or Belize or "Bosnia and Herzegovina" or Botswana or Brazil or Bulgaria or China or Colombia or Costa Rica or Cuba or Dominica or Dominican Republic or Equatorial Guinea or Ecuador or Fiji or Gabon or Georgia or Grenada or Guatemala or Guyana or Iraq or Jamaica or Jordan or Kazakhstan or Kosovo or Libya or Malaysia or Maldives or Marshall Islands or Mauritius or Mexico or Moldova or Montenegro or Namibia or North Macedonia or Palau or Paraguay or Peru or Russian Federation or Serbia or South Africa or Saint Lucia or "Saint Vincent and the Grenadines" or Suriname or Thailand or Tonga or Turkiye or Turkmenistan or Tuvalu)
**Subject Headings**			
**Embase (Ovid)**	exp *patient preference/ or exp *decision making/ or exp *patient decision making/ or exp *shared decision making/	exp *maternal health service/ or exp *prenatal care/ or exp *postnatal care/ or childbirth/ or exp *labor/ or maternal health	exp Afghanistan/ or exp Albania/ or exp Algeria/ or exp American Samoa/ or exp Angola/ or exp Argentina/ or exp Armenia/ or exp Azerbaijan/ or exp Bangladesh/ or exp Belize/ or exp Benin/ or exp Bhutan/ or exp Bolivia/ or exp "Bosnia and Herzegovina"/ or exp Botswana/ or exp Brazil/ or exp Bulgaria/ or exp Burkina Faso/ or exp Burundi/ or exp Cabo Verde/ or exp

		care or maternal healthcare or maternity care or<br>maternity health care or maternity healthcare	Cambodia/ or exp Cameroon/ or exp Central African Republic/ or exp Chad/ or exp China/ or exp Colombia/ or exp Comoros/ or exp Congo/ or exp "Democratic Republic of the Congo"/ or exp Costa Rica/ or exp Cote d'Ivoire/ or exp Cuba/ or exp Djibouti/ or exp Dominica/ or exp Dominican Republic/ or exp Ecuador/ or exp Egypt/ or exp El Salvador/ or exp Equatorial Guinea/ or exp Eritrea/ or exp Eswatini/ or exp Ethiopia/ or exp Fiji/ or exp Gabon/ or exp Gambia/ or exp "Georgia (Republic)"/ or exp Ghana/ or exp Grenada/ or exp Guatemala/ or exp Guinea/ or exp Equatorial Guinea/ or exp Guinea-Bissau/ or exp Guyana/ or exp Haiti/ or exp Honduras/ or exp India/ or exp Indonesia/ or exp Iran/ or exp Iraq/ or exp Jamaica/ or exp Jordan/ or exp Kazakhstan/ or exp Kenya/ or exp "Democratic People's Republic of Korea"/ or exp "Republic of Korea"/ or exp Korea/ or exp Kosovo/ or exp Kyrgyzstan/ or exp Lebanon/ or exp Lesotho/ or exp Liberia/ or exp Libya/ or exp Madagascar/ or exp Malawi/ or exp Malaysia/ or exp Maldives/ or exp Mali/ or exp Mauritania/ or exp Mauritius/ or exp Mexico/ or exp "Gulf of Mexico"/ or exp New Mexico/ or exp Micronesia/ or exp Moldova/ or exp Mongolia/ or exp Montenegro/ or exp Morocco/ or exp Mozambique/ or exp Myanmar/ or exp Namibia/ or exp Nepal/ or exp Nicaragua/ or exp Niger/ or exp Aspergillus niger/ or exp Nigeria/ or exp Pakistan/ or exp Palau/ or "Papua New Guinea".mp. or exp Papua New Guinea/ or Paraguay.mp. or exp Paraguay/ or exp Peru/ or exp Philippines/ or exp "Republic of Belarus"/ or exp "Republic of North Macedonia"/ or exp Russia/ or exp Rwanda/ or exp Saint Lucia/ or exp "Saint Vincent and the Grenadines"/ or exp Samoa/ or exp "Sao Tome and Principe"/ or exp Senegal/ or exp Serbia/ or exp Sierra Leone/ or exp Somalia/ or exp South Africa/ or exp South Sudan/ or exp Sri Lanka/ or exp Sudan/ or exp South Sudan/ or exp Suriname/ or exp Syria/ or exp

			Tajikistan/ or exp Tanzania/ or exp Thailand/ or exp Timor-Leste/ or exp Togo/ or exp Tonga/ or exp Tunisia/ or exp "Turkey (republic)"/ or exp Turkmenistan/ or exp Uganda/ or exp Ukraine/ or exp Uzbekistan/ or exp Vanuatu/ or exp Vietnam/ or exp Yemen/ or exp Zambia/ or exp Zimbabwe/
**Medline (Ovid)**	exp Patient Preference/ or preferences OR exp *Decision Making, Shared/	exp *maternal-child health services/ or exp *perinatal care/ or exp *prenatal care/ or exp *maternal health services/ or exp *labor, induced/ or exp *vaginal birth after cesarean/ or exp *Maternal Health/ or *birth setting/ or exp *natural childbirth/ or exp Postnatal Care/ or exp *Postpartum Period/	Developing Countries/ or exp Resource-Limited Settings/ or exp africa/ or exp africa, northern/ or exp "africa south of the sahara"/ or exp "Africa South of the Sahara"/ or exp Afghanistan/ or exp Albania/ or exp Algeria/ or exp American Samoa/ or exp Angola/ or exp Argentina/ or exp Armenia/ or exp Azerbaijan/ or exp Bangladesh/ or exp Belize/ or exp Benin/ or exp Bhutan/ or exp Bolivia/ or exp "Bosnia and Herzegovina"/ or exp Botswana/ or exp Brazil/ or exp Bulgaria/ or exp Burkina Faso/ or exp Burundi/ or exp Cabo Verde/ or exp Cambodia/ or exp Cameroon/ or exp Central African Republic/ or exp Chad/ or exp China/ or exp Colombia/ or exp Comoros/ or exp Congo/ or exp "Democratic Republic of the Congo"/ or exp Costa Rica/ or exp Cote d'Ivoire/ or exp Cuba/ or exp Djibouti/ or exp Dominica/ or exp Dominican Republic/ or exp Ecuador/ or exp Egypt/ or exp El Salvador/ or exp Equatorial Guinea/ or exp Eritrea/ or exp Eswatini/ or exp Ethiopia/ or exp Fiji/ or exp Gabon/ or exp Gambia/ or exp "Georgia (Republic)"/ or exp Ghana/ or exp Grenada/ or exp Guatemala/ or exp Guinea/ or exp Equatorial Guinea/ or exp Guinea-Bissau/ or exp Guyana/ or exp Haiti/ or exp Honduras/ or exp India/ or exp Indonesia/ or exp Iran/ or exp Iraq/ or exp Jamaica/ or exp Jordan/ or exp Kazakhstan/ or exp Kenya/ or exp "Democratic People's Republic of Korea"/ or exp "Republic of Korea"/ or exp Korea/ or exp Kosovo/ or exp Kyrgyzstan/ or exp Lebanon/ or exp Lesotho/ or exp Liberia/ or exp Libya/ or exp Madagascar/ or exp Malawi/ or exp Malaysia/ or exp Maldives/ or exp

			Mali/ or exp Mauritania/ or exp Mauritius/ or exp Mexico/ or exp "Gulf of Mexico"/ or exp New Mexico/ or exp Micronesia/ or exp Moldova/ or exp Mongolia/ or exp Montenegro/ or exp Morocco/ or exp Mozambique/ or exp Myanmar/ or exp Namibia/ or exp Nepal/ or exp Nicaragua/ or exp Niger/ or exp Aspergillus niger/ or exp Nigeria/ or exp Pakistan/ or exp Palau/ or exp Papua New Guinea/ or exp Paraguay/ or exp Peru/ or exp Philippines/ or exp "Republic of Belarus"/ or exp "Republic of North Macedonia"/ or exp Russia/ or exp Rwanda/ or exp Saint Lucia/ or exp "Saint Vincent and the Grenadines"/ or exp Samoa/ or exp "Sao Tome and Principe"/ or exp Senegal/ or exp Serbia/ or exp Sierra Leone/ or exp Somalia/ or exp South Africa/ or exp South Sudan/ or exp Sri Lanka/ or exp Sudan/ or exp South Sudan/ or exp Suriname/ or exp Syria/ or exp Tajikistan/ or exp Tanzania/ or exp Thailand/ or exp Timor-Leste/ or exp Togo/ or exp Tonga/ or exp Tunisia/ or exp Turkey/ or exp Turkmenistan/ or exp Uganda/ or exp Ukraine/ or exp Uzbekistan/ or exp Vanuatu/ or exp Vietnam/ or exp Yemen/ or exp Zambia/ or exp Zimbabwe/
PsycINFO	exp *preferences/ or exp *experimental design/or Forced Choice.mp. or patient ranking.mp. or discrete choice.mp.	exp Antepartum Period/ or exp Pregnancy/ or exp Prenatal Care/ or exp Health Care Utilization / or exp *health care utilization/ or exp Childrearing Practices/ or exp Birth/ or exp Postnatal Period/ or exp Obstetrics/ or exp Birth/ or exp Pregnancy/or exp Caesarean Birth or exp Health	exp Developing Countries/ or exp *Lower Income Level/ or exp *middle income level/ or exp *middle socioeconomic status/

		Care Utilization/ or *Health Care Delivery/	
CINAHL<br><br>(EBSCO)	(MH "Patient Preference") or (MH "Forced Choice Scaling") or (MM "Decision Making, Patient+") OR (MM "Decision Making, Shared") or (MH "Consumer Participation")	(MH "Maternal Health Services+") OR (MM "Prenatal Care") OR (MM "Pregnancy Care (Saba CCC)") OR OR (MH "Vaginal Birth+") OR (MH "Childbirth+") OR<br><br>(MH "Delivery Rooms") OR (MH "Hospital Units/OG/UT/LS") OR (MH "Perinatal Care") OR (MH "Prenatal Care") OR (MH "Postnatal Care") OR (MM "Alternative Birth Centers") OR (MM "Home Childbirth") OR (MM "Prepared Childbirth") OR (MH "Delivery Rooms+") OR (MH "Labor and Delivery Nursing") OR (MM "Labor and Delivery Nurses") OR (MH "Postnatal Care+") OR (MH "Postnatal Period+") OR (MM "Postpartum Care (Saba CCC)")	(MM "Developing Countries") OR (MM "Low and Middle Income Countries") OR (MM "Africa+") OR (MM "Africa South of the Sahara+") OR (MM "Africa, Western+") OR (MM "Africa, Southern+") OR (MM "Africa, Northern+") OR (MM "Africa, Eastern+") OR (MM "Africa, Central+") OR (MM "South Africa") OR “resource limited setting” OR "resource limited regions" OR "resource limited countries"

Scopus	Keywords and titles	Keywords and titles	Keywords and titles
Global Health	exp consumer preferences/ or exp willingness to pay/or consumer attitudes or decision making/ or exp decision analysis/ or exp decision support systems/	exp prenatal care/ or exp parturition/ or exp prenatal care/ or exp lactating women/ or exp pregnant women/ or exp women's health/	exp least developed countries/ or exp low income countries/ or exp lower-middle income countries/or exp Africa South of Sahara or exp afghanistan/ or exp angola/ or exp bangladesh/ or exp benin/ or exp bhutan/ or exp burkina faso/ or exp burundi/ or exp cambodia/ or exp central african republic/ or exp chad/ or exp comoros/ or exp congo democratic republic/ or exp djibouti/ or exp east timor/ or exp eritrea/ or exp ethiopia/ or exp gambia/ or exp guinea/ or exp guinea-bissau/ or exp haiti/ or exp kiribati/ or exp laos/ or exp lesotho/ or exp liberia/ or exp madagascar/ or exp malawi/ or exp mali/ or exp mauritania/ or exp mozambique/ or exp myanmar/ or exp nepal/ or exp niger/ or exp rwanda/ or exp "sao tome and principe"/ or exp senegal/ or exp sierra leone/ or exp solomon islands/ or exp somalia/ or exp south sudan/ or exp sudan/ or exp tanzania/ or exp togo/ or exp tuvalu/ or exp uganda/ or exp yemen/ or exp zambia/
Google scholar	(“discrete choice” | “stated preference” | Thurstone | conjoint | DCE)	(delivery | obstetric | antenatal | prenatal | postnatal | maternal | pregnant | "follow up")	(Developing | undeveloped |"low income" | "low-income*" | "middle income" | "middle-income" | "low resource" | “resource limited” | "middle resource" |"low-and middle- income" | "low and middle income" | "non-industrialised" |"third world")

Supplementary Table 2. Inclusion and exclusion criteria for review of preferences of women for maternal healthcare services in LMICs

PICO	Inclusion criteria	Exclusion criteria
Population	Women (15 years old and above)	Studies done among women healthcare provider
Intervention	Not applicable	Not applicable
Comparison	Not applicable	Not applicable
Outcome	Stated preference for maternal health services (ANC, obstetric care and PNC),	Revealed preference, satisfaction, acceptability, and utilisation
Type of Study	Primary observational studies using:<br>Discrete choice experiment (DCE)<br>Best Worst, Thurstone scale	Government reports, qualitative studies, case studies, case series, systematic reviews, conference presentations, commentaries
Context	Countries in LMICs	
Year	Studies later than 2000	
Language	Studies conducted in English	

Supplementary Table 3: Excluded reviews and reasons for exclusion after full-text review

S.N	Studies	Reasons for exclusion
**I.**	**Settings were not LMICs**	
1.	Abdulrahim (2021)[1]	High-income country (Scotland)
2.	Deverill (2010)[2]	High-income country (United Kingdom (UK))
3.	Fawsit (2017)[3]	High-income country (Ireland)
4.	Fawsit (2017)[4]	High-income country (Ireland)
5.	Gärtner (2015)[5]	High-income country (Netherlands)
6.	Howard (2014)[6]	High-income country (Australia)
7.	Hundley (2004)[7]	High-income country (Scotland)
8.	Hundley (2001)[8]	High-income country (Scotland)
9.	Rheindorf (2021)[9]	High-income country (Germany)
10.	Ride (2016)	High-income country (Australia)
11.	Scotland (2011)[10]	High-income country (Scotland)
**II.**	**The outcome was not maternal health services**	
12.	Adams (2016)[11]	The outcome was a willingness to pay for a new maternal nutrient supplement
13.	Brown (2018)[12]	The outcome was preferences for community-based health programs in Tanzania (chapter 5)
14.	Hanson (2005)[13]	The outcome was preferences for hospital quality in Zambia
15.	Morhason (2008)[14]	The outcome was the attitude and preferences of Nigerian antenatal women to social support during labour
16.	Nargesi (2021)[15]	The outcome was the preferences of Iranians to select the emergency department physician at the time of service delivery
17.	Ozdemir (2021)[16]	The outcome was parturients' Stated Preferences for Labor Analgesia: A Discrete Choice Experiment
18.	Umar (2018)[17]	The outcome was exploring the dis-utilities associated with attributes of disrespect and abuse during institutional birth
**III.**	**Method was not conjoint method**	
19.	Alcock (2015)[18]	Examining inequalities in uptake of maternal health care and choice of provider using binary logistic regression
20.	Aremu (2011)[19]	Patterns of delivery care utilization in Nigeria: a multilevel discrete choice analysis
21.	Aziz (2022)[20]	Determine childbirth method considering uncertainties, and patient preferences, using multivariate binary logistic regression analysis
22.	Bharadwaj (2020)[21]	Determinants of Place of Childbirth in Chamba District, Himachal Pradesh, India using binary logistic regression analysis
23.	Dehury (2021)[22]	The change of preference for location of childbirth for successive births using logistic regression analysis
24.	Deleault (2015)[23]	Treatment Decision Making in the Postpartum Period: Examining Women's Preferences and Perspectives using Problem-Solving Decision Making Scale.
25.	Edmonds (2012)[24]	Determinants of place of birth decisions in uncomplicated childbirth in Bangladesh: using binary logistic regression analysis
26.	Enuku (2022)[25]	Factors influencing preference for traditional birth attendant services compared to hospital care using analysis of variance (ANOVA).

27.	Ibrahim (2021)[26]	Preference of mode of delivery among women in the childbearing period in Egypt and factors affecting it using logistic regression analysis
28.	Kenya (2015)[27]	Estimating willingness to pay for maternal health services using multivariate regression analysis
29.	Konde (2011)[28]	Ugandan women's childbirth preferences using descriptive analysis.
30.	Kosan (2019)[29]	Delivery preferences of pregnant women: Do not underestimate the effect of friends and relatives using binary logistic regression analysis
31.	Mahdi (2010)[30]	A study on preference and practices of women regarding place of delivery using descriptive analysis.
32.	Mgdulwa (2017)[31]	Self-reported preference for a delivery place among women presenting for maternal care health services at a tertiary hospital in the eastern Cape province, south Africa using descriptive analysis.
**IV.**	**Qualitative studies**	
33.	Arije (2022)[32]	Attributes development for a discrete choice experiment on preferences in sexual and reproductive health services
34.	Atukunda (2020)[33]	Women's Choice to Deliver at Home: Understanding the Psychosocial and Cultural Factors Influencing Birthing Choices for Unskilled Home Delivery among Women in Southwestern Uganda
35.	Chiwire (2022)[34]	Identification and Prioritization of Attributes for a Discrete Choice Experiment Using the Nominal Group Technique
36.	Jacobs (2023)[35]	Understanding maternal choices and experiences of care by skilled providers: Voices of mothers who delivered at home in selected communities of Lusaka city, Zambia using a phenomenological case study design
37.	Pokhrel (2006)[36]	Commentary paper on Scaling up health interventions in resource-poor countries: what role does research in stated-preference framework play
**V.**	**Full text is not available**	
38.	Kachwaha (2019)[37]	Women's Stated Preferences for Conditional Cash Transfer Programs Focused on Maternal and Child Health and Nutrition in India (P22-012-19)
39.	Ternent (2007)[38]	Using Discrete Choice Experiments to Elicit Preferences for Maternal Health Care in Ghana

Supplementary Table 4. Attributes and levels and sample DCE scenarios for the preferences of women for maternal healthcare services in LMIC's.

1st author, year and setting	Attributes and levels	Sample DCE scenarios
Beam (2017)[39], Ethiopia	1. Distance to health facility: 30 minutes, 1 hour, 1½ hours, 2 hours, 3 hours.<br>2. Type of provider: Female doctor, Male doctor, Female nurse, Male nurse, Health extension workers<br>3. Provider attitude: Provider smiles, is kind and respectful and speaks softly, Provider does not smile, use a harsh tone, or harsh language.<br>4. Availability of medication and supplies: Drugs and medical equipment are always available, and Drugs and medical equipment not always available<br>5. Availability of transport: Free ambulance available: Free ambulance not available<br>6. Support persons: Family and friends allowed in the delivery room, Family and friends not allowed in the delivery room<br>7. "Cost (Cost of user charges, labour-related supplies, and non-ambulance transportation) No cost: 50 ETB, 100ETB, 200ETB, 300ETB	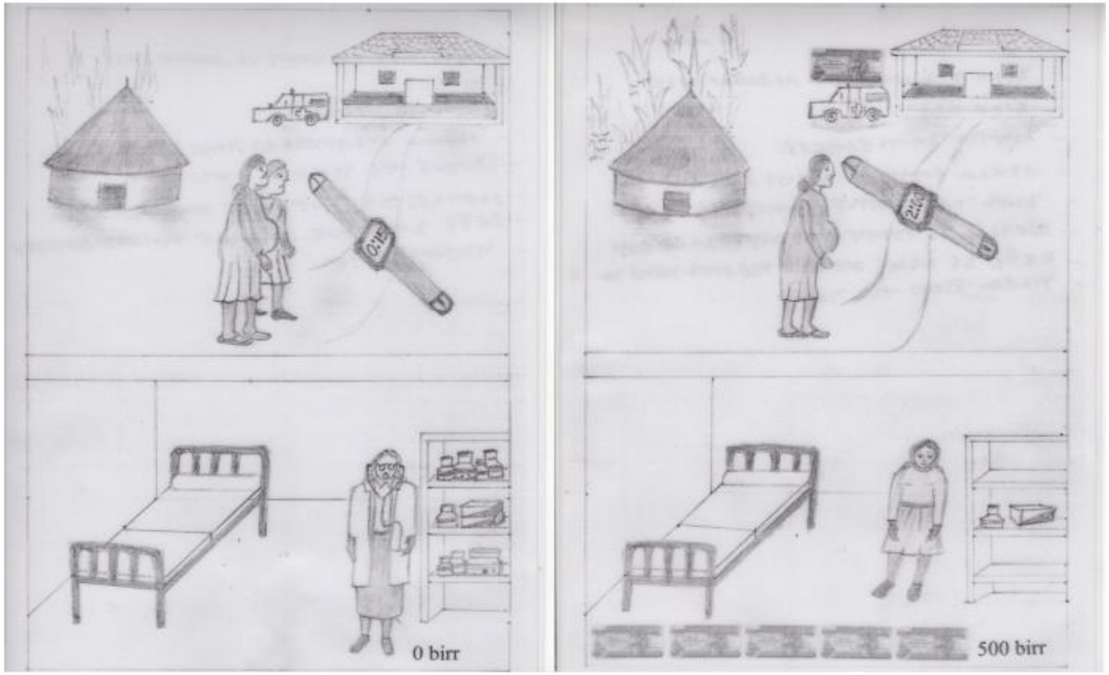

<table>
<tr>
<td>Camacho (2022)[40], Tanzania</td>
<td>1. Transport: Walk, Bicycle/motorbike, public transport (bus), Private car/taxi

2. Cleanliness: Not clean enough, Clean enough
3. Comfort Seats: Shaded waiting area, Drinking water, Toilet/washroom.

4. Content: Conversation, Conversation and physical examination, AND Conversation, physical examination, and birth preparedness education"

5. Staff attitude: Harsh and rude, Kind and friendly (respectful care)</td>
<td>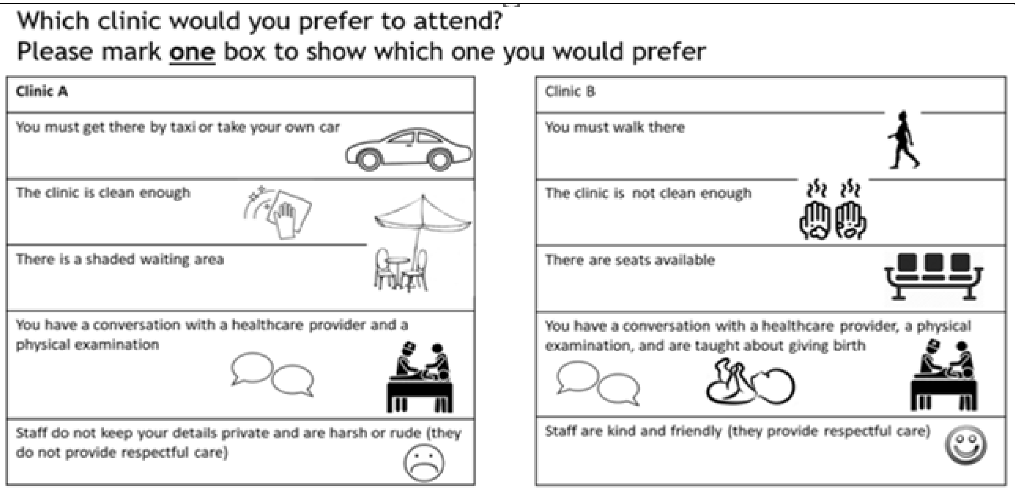
</td>
</tr>
<tr>
<td>Kruk (2010)[41], Ethiopia</td>
<td>1. Distance by foot:1/2 hour, 1 hour , 1 and 1/2 hours, 2 hours 3 hours

2. Transport: available, not available

3. Types of provider: Doctor, Nurse, health extension
4. Provider attitude: Provider smiles, listens carefully, Provider does not smile, does not listen carefully.
5. Drug and equipment: always available not always available
6. Cost: 3 ETB, 5 ETB, 10 ETB, 20 ETB, 30 ETB, 2000 Shillings, 3000 Shillings</td>
<td><table>
<tr><td></td><td>Health Center A</td><td>Health Center B</td><td>C</td></tr>
<tr><td>Distance</td><td>1/2 hour by foot</td><td>1 and 1/2 hours by foot</td><td>I do not want to go to either health center</td></tr>
<tr><td>Provider type</td><td>Doctor</td><td>Health extension worker</td><td></td></tr>
<tr><td>Provider attitude</td><td>Provider smiles, listens carefully</td><td>Provider does not smile, does not listen carefully</td><td></td></tr>
<tr><td>Availability of drugs and medical equipment</td><td>Drugs and medical equipment not always available</td><td>Drugs and medical equipment always available</td><td></td></tr>
<tr><td>Availability of transport</td><td>Transport not available</td><td>Transport not available</td><td></td></tr>
<tr><td>Total cost</td><td>Cost is 3 birr</td><td>10 Birr</td><td></td></tr>
</table></td>
</tr>
</table>

Kruk (2009)[42], Tanzania	1. Distance by foot:1/2 hour, 1 hour, 1 and 1/2 hours, 2 hours, 3 hours<br>2. Transport: available, not available<br>3. Types of providers: Doctor, Clinical officer, Nurse<br>4. Provider attitude: The provider smiles, and listens carefully, The Provider does not smile, and does not listen carefully.<br>5. Drug and equipment: always available not always available.<br>6. Cost: 250 Shillings, 500 Shillings, 1000 Shillings, 2000 Shillings, 3000 Shillings	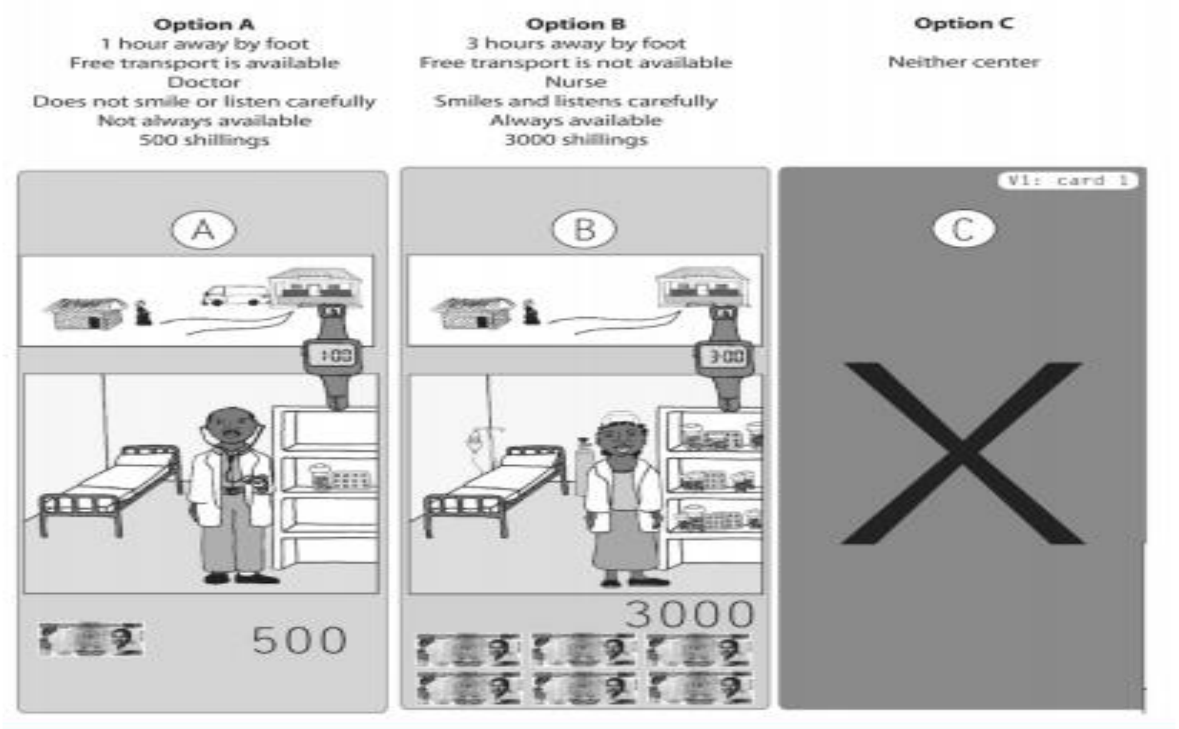

Kumar (2023)[43], Kenya	1. Information delivery: Health Facility, Individualized<br>2. Participants: Co-participate with Caregivers and provide information sheets for caregivers<br>3. Treatment option: 4 sessions for 1.5 hours, 8 sessions for 1.5 hours<br>4. Intervention delivery: CHV, Facility Nurses<br>5. Training: Vocational, Formal (Back to school)<br>6. Support: Peer support, Parenting skills<br>7. Services: Adolescent-friendly services, Combined with older mothers<br>8. Incentives: Transport:, KSh. 500, Food, Both	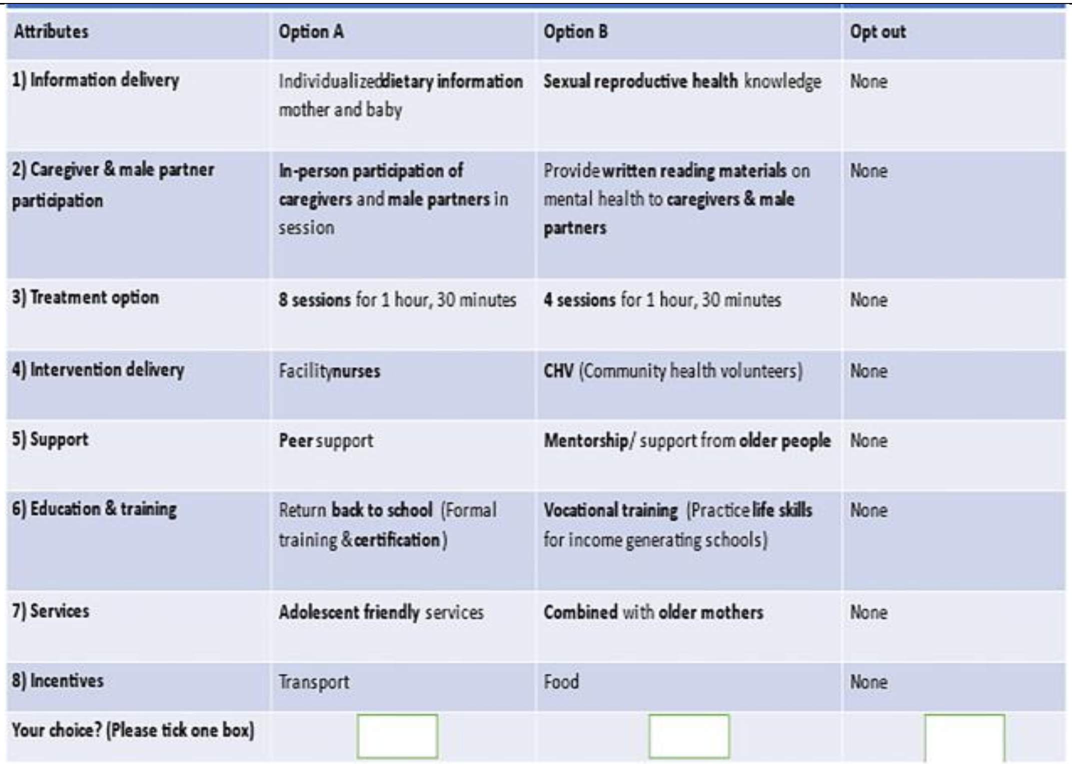

Attributes	Option A	Option B	Opt out
1) Information delivery	Individualized **dietary information** mother and baby	**Sexual reproductive health** knowledge	None
2) Caregiver & male partner participation	**In-person participation of caregivers** and **male partners** in session	Provide **written reading materials** on mental health to **caregivers & male partners**	None
3) Treatment option	**8 sessions** for 1 hour, 30 minutes	**4 sessions** for 1 hour, 30 minutes	None
4) Intervention delivery	Facility **nurses**	**CHV** (Community health volunteers)	None
5) Support	**Peer** support	**Mentorship**/ support from **older people**	None
6) Education & training	Return **back to school** (Formal training & **certification**)	**Vocational training** (Practice **life skills** for income generating schools)	None
7) Services	**Adolescent friendly** services	**Combined** with **older mothers**	None
8) Incentives	Transport	Food	None
Your choice? (Please tick one box)			

Larson (2015)[44], Tanzania	1. Medical equipment & drugs: Facility has modern equipment and drugs, Facility has poor equipment and shortage of drugs<br>2. Doctor's medical knowledge: Doctor has excellent medical knowledge, Doctor has basic medical knowledge<br>3. Doctor's attitude: Doctor treats me kindly, Doctor does not treat me kindly<br>4. Facility cleanliness & organization: The facility is clean and tidy, The facility is not clean and tidy<br>5. Privacy: I have privacy when I deliver, I don't have privacy when I deliver<br>6. Cost: TZS 2,000 TZS; 5,000 TZS; 10,000 TZS; 20,000 TZS; 30,000 TZS	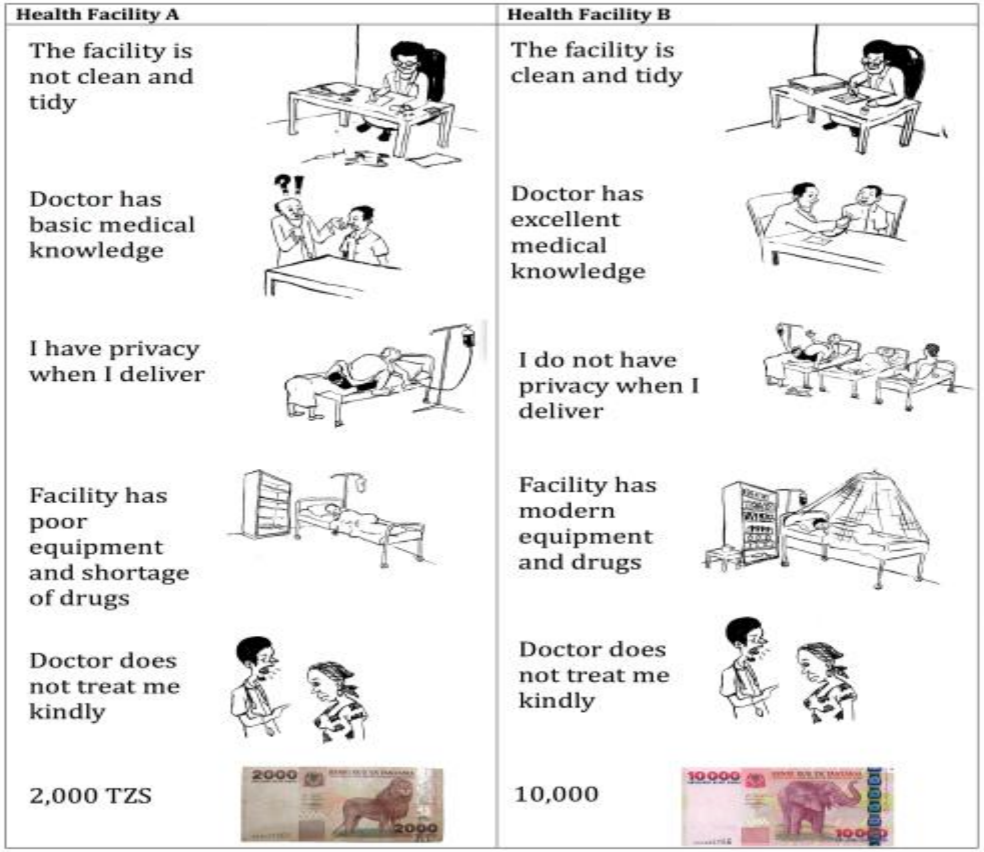

Mahumud (2018)[45], Bangladesh	1. Availability of Provider: Paramedic, Nurse, Female Doctor, Male Doctor<br>2. Provider Attitude: Polite, Rude<br>3. Price: No fee, Normal delivery (<600 BDT), Normal delivery (≤800 BDT)<br>4. Continuum of Maternal Healthcare (MHC) Services: No delivery service, Normal delivery + (ANC and PNC), Normal delivery + (ANC, PNC and Referral), Normal delivery + (ANC, PNC and Ambulance services for referral), and Normal delivery + (C-section, ANC, and PNC)<br>5. Availability of Drug: Brand drugs, Non-brand drugs, Uncertain or no drugs"<br>6. Availability of Diagnostic Services: Yes, No<br>7. Facility Environment Clean: Yes, No<br>8. Availability for complaints: No option, Comment box, Assigned person, Phone line<br>9. Waiting Times (in minutes): <60, 60–120, >120, None	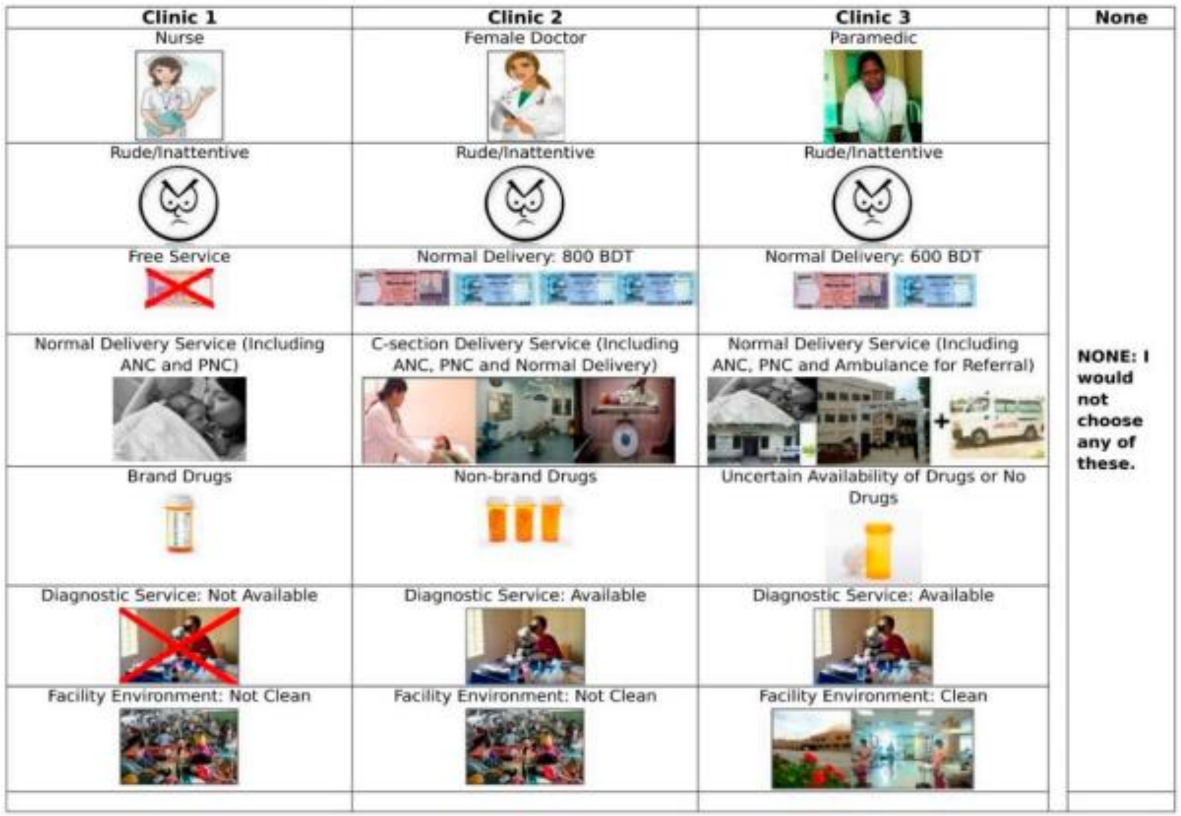
Mazzoni (2016)[46], Argentina	1. Possibility of scheduling the date of delivery (Yes/No)<br>2. Episiotomy (Yes/No)<br>3. Sexual function at 6 months postpartum (the same than before delivery/worse than before delivery"<br>4. Pain during delivery (mild/moderate/severe)<br>5. Recovery after delivery (less than 1 week/between 1 and 2 weeks/more than 2 weeks)	**No picture available**

<table>
<tr>
<td>Oluoch-Aridi (2020)[47], Kenya</td>
<td>1. Quality of clinical services at the health facility: Good quality of clinical services, Bad quality of clinical services"
2. Attitude of healthcare workers: Kind and supportive: Unkind and unsupportive
3. Availability of medical equipment and supplies: Available and not available
4. Distance: health facility is close to the residence: Health facility is far from residence
5. Cleanliness of the health facility: Clean, Dirty "
6. Cost of delivery service: 3000; 5000; 8000</td>
<td>
<table>
<tr><th></th><th>Clinic A</th><th>Clinic B</th><th>Option C</th></tr>
<tr><td>Treatment at health facility</td><td>Bad</td><td>Good</td><td>(None of the two health facilities- home delivery)</td></tr>
<tr><td>Cleanliness</td><td>Dirty</td><td>Clean</td><td></td></tr>
<tr><td>Attitude of Health worker</td><td>Unkind/Not Supportive</td><td>Kind & Supportive</td><td></td></tr>
<tr><td>Medical equipment & Drugs</td><td>Always available</td><td>Always available</td><td></td></tr>
<tr><td>Distance to facility</td><td>Below 1 hour by transport</td><td>More than 1 hour by transport</td><td></td></tr>
<tr><td>Cost</td><td>3000</td><td>5000</td><td>□</td></tr>
</table>
</td>
</tr>
<tr>
<td>Oluoch-Aridi (2020)[48], Kenya</td>
<td>1. Quality of clinical services at the health facility: Good quality of clinical services, Bad quality of clinical services"
2. Attitude of healthcare workers: Kind and supportive: Unkind and unsupportive
3. Availability of medical equipment and supplies: Available and not available
4. Distance: health facility is close to the residence: Health facility is far from residence
5. Cleanliness of the health facility: Clean, Dirty "
6. Cost of delivery service: 3000; 5000; 8000</td>
<td>
<table>
<tr><th>Attribute</th><th>Health facility A</th><th>Health facility B</th><th>Option C</th></tr>
<tr><td>Quality of clinical care during delivery</td><td>Good quality</td><td>Bad quality</td><td>(None of the two health facilities—home delivery)</td></tr>
<tr><td>Attitude of healthcare workers</td><td>Kind and supportive attitude</td><td>Unkind attitude</td><td></td></tr>
<tr><td>Cost of delivery services</td><td>Ksh3000</td><td>Ksh5000</td><td></td></tr>
<tr><td>Availability of equipment and supplies</td><td>Equipment supplies not available</td><td>Equipment and supplies available</td><td></td></tr>
<tr><td>Distance to health facility</td><td>Facility is close to home</td><td>Facility is far from home</td><td></td></tr>
<tr><td>Availability of referral health services</td><td>Referral services available</td><td>Referral services unavailable</td><td></td></tr>
<tr><td>Your choice (tick only one)</td><td>□</td><td>□</td><td>□</td></tr>
</table>
</td>
</tr>
</table>

Paczkowski (2012)[49], Ethiopia	1. Distance by foot:1/2 hour, 1 hour , 1 and 1/2 hours, 2 hours 3 hours<br>2. Transport: avialbel, not avilable<br>3. Types of provider: Doctor, Nurse, health extension<br>4. Provider attitude: Provider smiles, listens carefully, Provider does not smile, does not listen carefully.<br>5. Drug and equipment: always available not always available.<br>6. Cost: 3 ETB, 5 ETB, 10 ETB, 20 ETB, 30 ETB,	(blank) / Health Center A / Health Center B / C<br>Distance / 1/2 hour by foot / 1 and 1/2 hours by foot / I do not want to go to either health center<br>Provider type / Doctor / Health extension worker<br>Provider attitude / Provider smiles, listens carefully / Provider does not smile, does not listen carefully<br>Availability of drugs and medical equipment / Drugs and medical equipment not always available / Drugs and medical equipment always available<br>Availability of transport / Transport not available / Transport not available<br>Total cost / Cost is 3 birr / 10 Birr
Rajasulochana (2016)[50], India	1. Waiting Time Between Arrival and Seen by Health Care Personne: 90 minutes, 45 minutes, 15 minutes,<br>2. Cleanliness of Wards and Toilets: Low, Moderate, High<br>3. Privacy Maintained during Physical Examination: Never, Sometimes, Always<br>4. Ward Visits by Specialists (O&G and Paediatrician): O&G only visits once in two days, O&G only visits once a day, Both O&G and paediatrician visit once a daily".<br>5. Attitude of Health Care Personnel: Negligent and rude, Formal, and business-like, W[50 51]arm, and caring"	**Choice 2** / Hospital A / Hospital B<br>**Waiting time between arrival and seen by health care personnel** / 15 minutes / 90 minutes<br>**Cleanliness of Ward and Toilet** / Moderate / High<br>**Privacy maintained during physical examination** / Always / Never<br>**Ward visits by Specialists (O&G and Pediatrician)** / O&G only visits once in two days / O&G only visits once a day<br>**Attitude of Health care personnel** / Negligent and rude / Formal and business-like<br>**Which hospital would you prefer (*Tick one box only*)?** / Prefer Hospital A [ ] / Prefer Hospital B [ ]

Rijsbergen (2013)[51], Tanzania	1. Provider: Nurse/midwife, Doctor<br>2. Provider attitude: Does not smile and listen carefully, Smiles, and listens carefully.<br>3. Distance: 0 h walking, 2 h walking"<br>4. Cost of service: 1 0000 TZS, 3,000 TZS, 6,000 TZS, 9,000 TZS"<br>5. Technical quality: Equipment and drugs not always available, Equipment and drugs always available	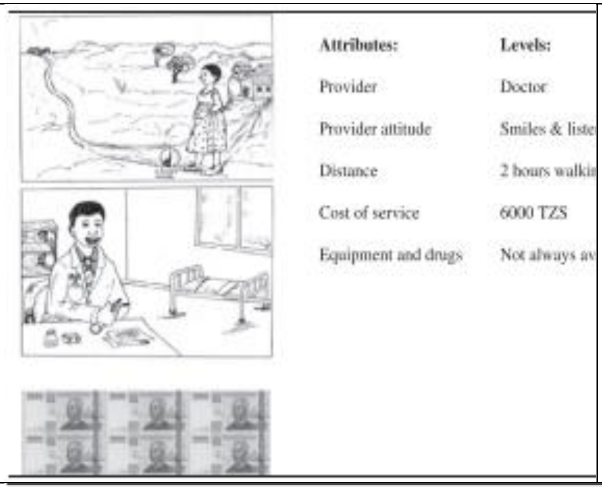<br><br>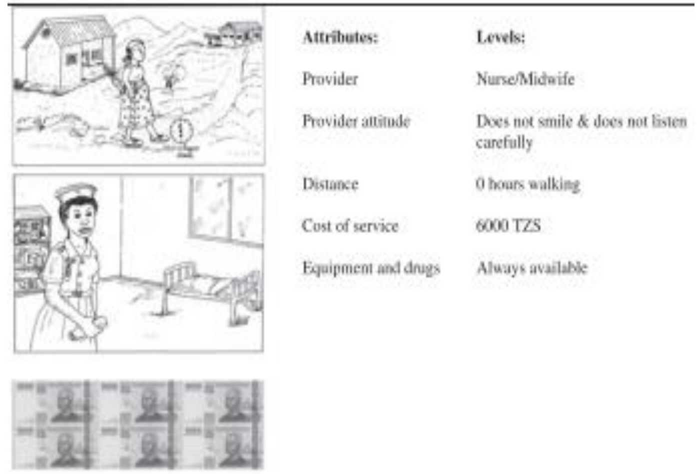
Umar (2023)[52], Nigeria	1. Failure to meet standards of care: Lack of informed consent and confidentiality: Physical examinations and procedures, Neglect and abandonment, Meeting professional standards of care:<br>2. Rapport with providers: Poor communication: Lack of supportive care: Loss of autonomy: Good rapport with providers:<br>3. Health system constraints: Staffing constraints, Drugs and supply constraints, Poor facility culture, good health system conditions<br>4. Physical and verbal abuse: Physical and verbal abuse, No physical and verbal abuse:<br>5. Sexual abuse: Sexual abuse: No sexual abuse:<br>6. Stigma and discrimination: Discrimination, No discrimination"	**Hospital A** / **Hospital B**<br>Hospital A: Birth attendant may not ask for your permission before performing any medical examination or procedure and may discuss your personal information openly with others. / Hospital B: Birth attendant may not give you pain relief as necessary (eg, during examination) or for the stitching of episiotomy.<br>Hospital A: Birth attendant may not explain what will happen to you or your baby and may not encourage you to ask questions or answer your questions. / Hospital B: Birth attendant will not empathise or show genuine interest in your well-being.<br>Hospital A: Qualified birth attendant not available to assist with your delivery. / Hospital B: Drugs and supplies needed for delivery not available.<br>Hospital A: Birth attendant may hit, slap or put restraints on you, shout or insult you. / Hospital B: Birth attendant will not hit, slap or put restraints on you and will not shout or insult you.<br>Hospital A: Birth attendant may touch your body parts or private parts inappropriately. / Hospital B: Birth attendant will not touch your body parts or private inappropriately.<br>Hospital A: Birth attendant may discriminate against you because you are poor, from a village, not educated or because of your religion, tribe or disease condition. / Hospital B: Birth attendant will not discriminate against you because you are poor, from a village, not educated or because of your religion, tribe or disease condition.<br>I will deliver at home
	1. Test procedure: Invasive, Non-invasive	**No picture available**

Wu (2023)[53], China	2. Time to wait for results: 1 week, 2 weeks, 3 weeks.<br>3. Detection rate: 94%, 96%, 98%, 100%.<br>4. Miscarriage: 3%, 4%, 5%.<br>5. Test cost: RMB$0, $2000, RMB$4000, RMB$6000, RMB$8000	

Supplementary Table 5. Dimensions of the included attributes for women's stated preferences for maternal healthcare services in LMICs (n=91).

**Accessibility (n=12)**	**Availability (n=27)**	**Accommodation (9)**	**Affordability (10)**	**Acceptability (33)**
• Distance or travel time to health facility (n=7)<br>• Transport method (n=5)	• Medical equipment & drugs (n=9)<br>• Types of health care provider (n=9)<br>• Content of care/procedure/service (n=3)<br>• Health system constraints (n=1)<br>• Doctor's medical knowledge (n=1)<br>• Treatment options (n=1)<br>• Test procedure type (n=1)<br>• Training and education (n=1)<br>• Incentives (n=1)	• Waiting time (n=3)<br>• Possibility of scheduling (n=1)<br>• Support persons allowed (n=2)<br>• Comfort waiting seats (n=1)<br>• Availability for complaints (n=1)<br>• Types of information delivery system (n=1)	Cost of services (n=10)	• Provider's attitude and report (n=12)<br>• Cleanliness (n=6)<br>• Quality of clinical services (n=3)<br>• Privacy (n=2)<br>• Participation of clients (n=1)<br>• Sexual, physical, and verbal abuse (n=2)<br>• Stigma and discrimination (n=1)<br>• Detection rate (n=1)<br>• Episiotomy (n=1)<br>• Sexual function (n=1)<br>• Pain during delivery (n=1)<br>• Miscarriage (n=1)<br>• Recovery after delivery (n=1)

Supplementary Table 6. The relative importance of attributes for women's stated preferences for maternal healthcare services in LMICs.

First author (year), country	Study period	Participants and sample size	Preferences of Maternal Health Care Service	The two most important attributes (Dimensions of access)	Least important attribute (Dimensions of access)
Beam (2017)[39], Ethiopia	September–October 2015	Women who were expecting a child or had a child less than two years old (n=108)	Delivery care	1. Availability of medications and supplies (availability)<br>2. Healthcare facilities that allowed support persons into the delivery room (accommodation)	Distance to the health facility (accessibility)
Camacho (2022)[40], Tanzania	2020	Pregnant women aged over 18 years (n=254)	ANC	1. Staff attitude (acceptability)<br>2. Waking distance (accessibility)	Cleanliness (acceptability)
Kruk (2010)[41], Ethiopia	May and August 2007	Women who had a child between the ages of 3 and 24 months (n=1006)	Delivery care	1. Availability of drugs and equipment (availability)<br>2. Provider attitude (acceptability)	Travel time (accessibility)
Kruk (2009)[42], Tanzania	June to mid-July 2007	Have delivery in the previous 5 years (n=1205)	Delivery care	1. Provider attitude (acceptability)<br>2. Availability of drugs and medical equipment (availability)	Travel time (accessibility)
Kumar (2023)[43], Kenya		Pregnant adolescents age from 14-18 (n=171)	ANC	1. Service delivery mode (availability)<br>2. Support type (availability)	Information delivery (accommodation)
Larson (2015)[44], Tanzania	February and April, 2012.	Women who were at least 15 years of age and delivered between six weeks and one year before the interview (n=2,950)	Delivery care	1. Doctor's attitude (acceptability)<br>2. Doctors' medical knowledge (availability)	Cost (affordability)
Mahumud (2018)[45], Bangladesh	June–July 2014	Women who delivered a baby in the past two years (n=421)	Delivery care	1. Availability of continuum of maternal healthcare (availability)<br>2. Availability of brand drugs (availability)	Price for service (affordability)
Mazzoni (2016)[46], Argentina (public hospital)	October 2010 and September 2011	Nulliparous, 32+ weeks gestation, aged 18 to 35 years who came for ANC in a public hospital (n=199).	Delivery care	1. Sexual function at 6 months of postpartum (acceptability).<br>2. Recovery after delivery (acceptability)	Possibility of scheduling childbirth (accommodation)

Mazzoni (2016)[46], Argentina (private hospital)	October 2010 and September 2011	Nulliparous, 32+ weeks gestation, aged 18 to 35 years who came for ANC in a private hospital (n=183)	Delivery care	1. Sexual function at 6 months (acceptability)<br>2. Pain during delivery (acceptability)	Possibility of scheduling childbirth accommodation)
Oluoch-Aridi (2020)[47], Kenya (peri-urban),	August and September 2017	Reproductive age (18–49 years) women who were delivered in the last five years (n=411).	Delivery care	1. Healthcare facility cleanliness (acceptability)<br>2. Availability of medical equipment and drugs (availability)	Cost of delivery service (affordability)
Oluoch-Aridi (2020)[48], Kenya (rural)	2017	Women aged 18–49 years who had delivered within the last 6 weeks (n = 466)	Delivery care	1. Attitude of healthcare workers (acceptability)<br>2. Availability of medical equipment and drugs (availability)	Cost of delivery service (affordability)
Paczkowski (2012)[49], Ethiopia (Depression)	May and August 2007	Highly Depressive and Had a child between the ages of 3 and 24 months	Delivery care	1. Provider attitude (acceptability)<br>2. Health care provider type (availability)	Distance (accessibility)
Paczkowski (2012)[49], Ethiopia (PTSD)	May and August 2007	PTSD and had a child between the ages of 3 and 24 months	Delivery care	1. Provider attitude (acceptability)<br>2. Health care provider type (availability)	Distance (accessibility)
Rajasulochana (2016)[50], India	November 2012 and March 2013	Expectant mothers who had come for antenatal check-ups	Delivery care	1. Ward visits by specialists (availability)<br>2. Cleanliness of wards and toilets (acceptability)	Waiting time (accommodation)
Rijsbergen (2013)[51], Tanzania	June 2010 and September 2010	Women who gave birth to at least one child in the last 5 years	Delivery care	1. Availability of equipment and drugs (availability)<br>2. Attitude of healthcare personnel (acceptability)	Types of healthcare providers (availability)
Umar (2023)[52], Nigeria	March-August 2018	Women who had recently delivered in a health facility	Delivery care	1. Good health system conditions (acceptability)<br>2. Absence of sexual abuse (acceptability)	Failure to meet the standard of care (acceptability)
Wu (2023)[53], China	January 2018, April 2018	High-risk pregnancy Attending the first visit for prenatal diagnostic consultation; gestational period no more than 20 weeks, 18 years old;	ANC	1. Test procedure (availability)<br>2. High detection rate (acceptability)	Time to wait for results (accommodation)

Hint: The five dimensions of access: accessibility, availability, accommodation, affordability and acceptability. ANC: antenatal care

Supplementary Table 7. Quality assessment of the included studies for women's stated preferences for maternal healthcare services in LMICs.

**Studies**	**Items**																														**The overall quality of the review**
	1. Well-defined research question			2. Attributes and levels of choice supported by evidence			3. Appropriate construction of tasks			**4.** Experimental design justified and evaluated			5. Appropriate Preferences elicit			6. Appropriate design of data collection instrument			7. Appropriate data collection plan			8. Appropriate statistical analyses			9. Valid results and conclusions			10. Clear, concise, and complete study presentation			
	**1.1**	**1.2**	**1.3**	**2.1**	**2.2**	**2.3**	**3.1**	**3.2**	**3.3**	**4.1**	**4.2**	**4.3**	**5.1**	**5.2**	**5.3**	**6.1**	**6.2**	**6.3**	**7.1**	**7.2**	**7.3**	**8.1**	**8.2**	**8.3**	**9.1**	**9.2**	**9.3**	**10.1**	**10.2**	**10.3**	
Beam (2017)	Y	Y	Y	Y	Y	Y	Y	Y	Y	Y	Y	Y	Y	Y	N	Y	Y	Y	Y	Y	Y	Y	Y	Y	Y	Y	Y	Y	Y	Y	Good
Camacho (2022)	Y	Y	Y	Y	Y	Y	Y	Y	Y	Y	Y	Y	Y	Y	N	Y	Y	Y	Y	Y	Y	Y	Y	Y	Y	Y	Y	Y	Y	Y	Good
Kruk (2010)	Y	Y	Y	Y	Y	Y	Y	Y	Y	Y	Y	Y	Y	Y	N	Y	Y	Y	Y	Y	Y	Y	Y	Y	Y	Y	Y	Y	Y	Y	Good
Kruk (2009)	Y	Y	Y	Y	Y	Y	Y	Y	Y	Y	Y	Y	Y	Y	N	Y	Y	Y	Y	Y	Y	Y	Y	Y	Y	Y	Y	Y	Y	Y	Good
Kumar (2023)	Y	Y	Y	Y	Y	Y	Y	Y	Y	Y	Y	Y	Y	Y	N	Y	Y	N	Y	Y	Y	Y	Y	Y	Y	Y	Y	Y	Y	Y	Good
Larson (2015)	Y	Y	Y	Y	Y	Y	Y	Y	Y	Y	Y	Y	Y	Y	N	Y	Y	Y	Y	Y	Y	Y	Y	Y	Y	Y	Y	Y	Y	Y	Good
Mahumud (2019)	Y	Y	Y	Y	Y	Y	N	Y	Y	N	Y	Y	Y	Y	Y	Y	Y	Y	Y	Y	Y	Y	Y	Y	Y	Y	Y	Y	Y	Y	Good
Mazzoni (2016)	Y	Y	Y	Y	Y	Y	N	Y	Y	N	N	Y	Y	Y	N	N	Y	N	Y	Y	Y	Y	N	Y	Y	Y	Y	N	Y	Y	moderate
Oluoch-Aridi (2020)	Y	Y	Y	Y	Y	Y	Y	Y	Y	Y	Y	Y	Y	Y	Y	Y	Y	Y	Y	Y	Y	Y	Y	Y	Y	Y	Y	Y	Y	Y	Good
Oluoch-Aridi (2020)	Y	Y	Y	Y	Y	Y	Y	Y	Y	Y	Y	Y	Y	Y	Y	Y	Y	NS	Y	Y	Y	Y	Y	Y	Y	Y	Y	Y	Y	Y	Good
Paczkowski (2012)	Y	Y	Y	Y	N	Y	Y	Y	Y	Y	Y	Y	Y	Y	N	Y	Y	Y	Y	Y	Y	Y	Y	Y	Y	Y	Y	Y	Y	Y	Good
Rajasulochana (2016)	Y	Y	Y	Y	Y	Y	Y	Y	Y	Y	Y	Y	Y	Y	N	N	Y	N	Y	Y	Y	Y	Y	Y	Y	Y	Y	Y	Y	Y	Good
Rijsbergen (2013)	Y	Y	Y	Y	Y	Y	Y	Y	N		Y	Y	Y	Y	N	N	Y	Y	Y	Y	Y	Y	Y	Y	Y	Y	Y	Y	Y	Y	Good
Umar (2020)	Y	Y	Y	Y	Y	Y	Y	Y	N	Y	Y	Y	Y	Y	Y	Y	Y	N	Y	Y	Y	Y	Y	Y	Y	Y	Y	Y	Y	Y	Good
Wu L (2023)	Y	Y	Y	Y	Y	Y	Y	Y	Y	Y	Y	Y	Y	Y	N	N	Y	N	Y	Y	Y	Y	Y	Y	Y	Y	Y	Y	Y	Y	Good

Note: Y: Yes; N: No.

Overall quality; Good: 25-30 points, Moderate: 20-25 points, Low: less than 20 points

A checklist for conjoint analysis applications in health care.

1. Was a well-defined research question stated and is conjoint analysis an appropriate method for answering it?

    1.1 Were a well-defined research question and a testable hypothesis articulated?

    1.2 Was the study perspective described, and was the study placed in a particular decision-making or policy context?

    1.3 What is the rationale for using conjoint analysis to answer the research question?

2. Was the choice of attributes and levels supported by evidence?

    2.1 Was attribute identification supported by evidence (literature reviews, focus groups, or other scientific methods)?

    2.2 Was attribute selection justified and consistent with the theory?

    2.3 Was level selection for each attribute justified by the evidence and consistent with the study perspective and hypothesis?

3. Was the construction of tasks appropriate?

    3.1 Was the number of attributes in each conjoint task justified (that is, full or partial profile)?

    3.2 Was the number of profiles in each conjoint task justified?

    3.3 Was (should) an opt-out or a status-quo alternative (be) included?

4. Was the choice of experimental design justified and evaluated?

    4.1 Was the choice of experimental design justified? Were alternative experimental designs considered?

    4.2 Were the properties of the experimental design evaluated?

    4.3 Was the number of conjoint tasks included in the data-collection instrument appropriate?

5. Were preferences elicited appropriately, given the research question?

    5.1 Was there sufficient motivation and explanation of conjoint tasks?

    5.2 Was an appropriate elicitation format (that is, rating, ranking, or choice) used? Did (should) the elicitation format allow for indifference?

    5.3 In addition to preference elicitation, did the conjoint tasks include other qualifying questions (for example, strength of preference,

confidence in response, and other methods)?

6. Was the data collection instrument designed appropriately?

6.1 Was appropriate respondent information collected (such as socio-demographic, attitudinal, health history or status, and treatment experience)?

6.2 Were the attributes and levels defined, and was any contextual information provided?

6.3 Was the level of burden of the data-collection instrument appropriate? Were respondents encouraged and motivated?

7. Was the data-collection plan appropriate?

7.1 Was the sampling strategy justified (for example, sample size, stratification, and recruitment)?

7.2 Was the mode of administration justified and appropriate (for example, face-to-face, pen-and-paper, web-based)?

7.3 Were ethical considerations addressed (for example, recruitment, information and/or consent, compensation)?

8. Were statistical analyses and model estimations appropriate?

8.1 Were respondent characteristics examined and tested?

8.2 Was the quality of the responses examined (for example, rationality, validity, reliability)?

8.3 Was model estimation conducted appropriately? Were issues of clustering and subgroups handled appropriately?

9. Were the results and conclusions valid?

9.1 Did study results reflect testable hypotheses and account for statistical uncertainty?

9.2 Were study conclusions supported by the evidence and compared with existing findings in the literature?

9.3 Were study limitations and generalizability adequately discussed?

10. Was the study presentation clear, concise, and complete?

10.1 Was study importance and research context adequately motivated?

10.2 Were the study data-collection instruments and methods described?

10.3 Were the study implications clearly stated and understandable to a wide audience?

Supplementary Table 8. The DCE methods for the stated preferences of women for maternal healthcare services in LMICs.

Studies	Total possible scenarios /choice tasks	No. of task /respon dent	Block design no.	No. of choice option per task without opt-out	Opt-out choice included	Number of attributes per option	Minimum and maximum label value (min, max)	Methods of analysis
Beam (2017)[39], Ethiopia		10	No	2	Yes	7	2,5	Multinomial logistic
Camacho (2022)[40], Tanzania	192	12	2	2	yes	5	2,4	Mixed logit model
Kruk (2010)[41], Ethiopia	600	8	No	2	yes	6	2,5	Hierarchical Bayesian
Kruk (2009)[42], Tanzania	600	8	5	2	yes	6	2,5	Hierarchical Bayesian
Kumar (2023)[43], Kenya		10	No	2	yes	8	2,3	Mixed multinomial logit
Larson (2015)[44], Tanzania	160	9	No	2	yes	6	2,5	Mixed multinomial logit
Mahumud (2018)[45], Bangladesh	-	12	No	3	yes	9	2,5	Hierarchical Bayesian/on Multinomial Logit
Mazzoni (2016)[46], Argentina (public hospital)	-	14	No	2	yes	5	2,3	Conditional logit
Mazzoni (2016)[46], Argentina (private hospital)	-	14	No	2	yes	5	2,3	Conditional logit
Oluoch-Aridi (2020)[47], Kenya (peri-urban),	96	8	2	2	yes	6	2,3	Mixed logit model
Oluoch-Aridi (2020)[48], Kenya (rural)	96	8	2	2	yes	6	2,3	Mixed logit model
Paczkowski (2012)[49], Ethiopia (Depression)	-	8	No	2	yes	6	2,5	Hierarchical Bayesian
Paczkowski (2012)[49], Ethiopia (PTSD)	-	8	No	2	Yes	6	2,5	Hierarchial Bayesian
Rajasulochana (2016)[50], India	243	9	3	2	Yes	5	3,3	Conditional logit model
Rijsbergen (2013)[51], Tanzania	64	8	No	3	No	5	2,4	Multinomial probit (MNP) regression
Umar (2023)[52], Nigeria	729	16	No	3	No	6	2,4	Mixed multinomial logit model
Wu (2023)[53], China	360	12	No	2	Yes	5	2,5	Conditional logistic regression

Supplementary Table 9: Sample standard scripts used for the study of preferences of women for maternal healthcare services in LMICs

1st author, year and setting	Sample standard scripts
Kruk (2009)[42], Tanzania	*"In this section, I will show you 8 cards. Each card describes 2 possible health centers. Imagine that you are deciding where to deliver your next baby. Please tell us which of the 2 centers you would prefer to go to for your delivery. You can decide not to use either of them. There are no right or wrong answers to these questions. We are only interested in learning about what is important to you about the health center"*
Oluoch-Aridi (2020)[47, 48], Kenya	*"Imagine that you are pregnant, and you are given a choice between the following two health facilities to deliver your baby in. Which one would you prefer? Facility A or Facility B? You also have an option of choosing none of the two health facilities as Option C. This implies delivering your baby at home. There are no right or wrong answers"*
Beam (2017)[39], Ethiopia	*"Imagine you are deciding where you will deliver your next baby. You are asked to choose between facility A, facility B or neither facility. Facility A has…. and facility B has… Which one you would prefer to go to for your delivery*
Camacho (2022)[40], Tanzania	*"Which clinic would you prefer to attend? Please mark one box to show which one would prefer"[38].*

Supplementary Table 10. Preference heterogeneity/Interactions with sociodemographic and/or disease-specific characteristics

First Author (Year), country	Maternal age	Education status	Resident	Marital status	Ethnicity	Parity	Economic status	Media Access	Distance to health facilities	Empowerment status	Complications in previous pregnancy	Depression	Post-traumatic stress disorder (PTSD)
Beam (2017)[39], Ethiopia							•			•			
Camacho (2022)[40], Tanzania		•	•			•							
Larson (2015)[44], Tanzania		•				•	•	•					
Mahumud (2018)[45], Bangladesh			•										
Oluoch-Aridi (2020)[47], Kenya (peri-urban),	•	•		•			•						
Oluoch-Aridi (2020)[48], Kenya (rural)	•	•		•			•						
Paczkowski (2012)[49], Ethiopia												•	•
Rajasulochana (2016)[50], India							•		•	•	•		
Umar (2023)[52], Nigeria	•	•			•		•						

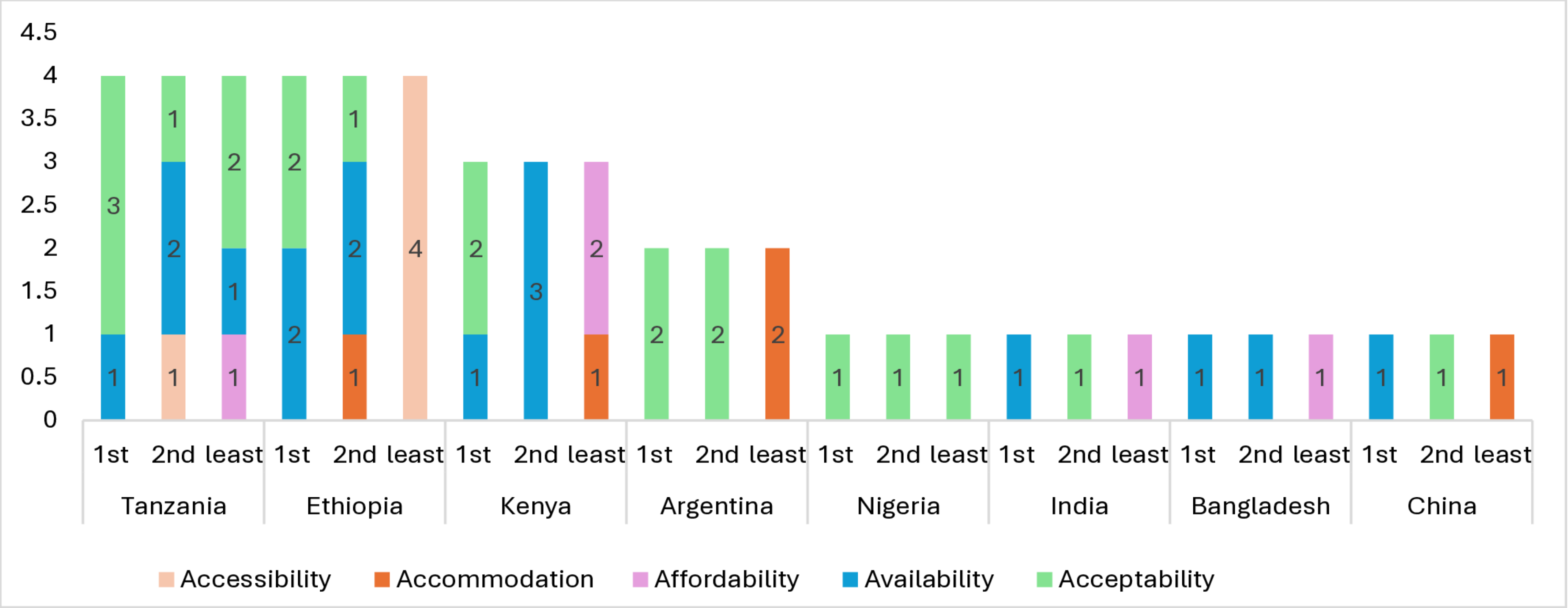

Supplementary Figure 1. The geographical distribution of stated preferences among women for maternal health services.

Reference

1. Abdulrahim B, Scotland G, Bhattacharya S, et al. Assessing couples' preferences for fresh or frozen embryo transfer: a discrete choice experiment. *Human Reproduction* 2021;36(11):2891-903. doi: doi:
2. Deverill M, Lancsar E, Snaith VBA, et al. Antenatal care for first time mothers: a discrete choice experiment of women's views on alternative packages of care. *European Journal of Obstetrics & Gynecology and Reproductive Biology* 2010;151(1):33-37. doi: doi:
3. Fawsitt CG, Bourke J, Greene RA, et al. What do women want? Valuing women's preferences and estimating demand for alternative models of maternity care using a discrete choice experiment. *Health Policy* 2017;121(11):1154-60. doi: doi:
4. Fawsitt CG, Bourke J, Lutomski JE, et al. What women want: exploring pregnant women's preferences for alternative models of maternity care. *Health policy* 2017;121(1):66-74. doi: doi:
5. Gärtner FR, de Bekker-Grob EW, Stiggelbout AM, et al. Calculating preference weights for the labor and delivery index: A discrete choice experiment on women's birth experiences. *Value in Health* 2015;18(6):856-64. doi: doi:
6. Howard K, Gerard K, Adelson P, et al. Women's preferences for inpatient and outpatient priming for labour induction: a discrete choice experim ent. *BMC health services research* 2014;14(1):1-11. doi: doi:
7. Hundley V, Ryan M. Are women's expectations and preferences for intrapartum care affected by the model of care on offer? *BJOG: An International Journal of Obstetrics & Gynaecology* 2004;111(6):550-60. doi: doi:
8. Hundley V, Ryan M, Graham W. Assessing women's preferences for intrapartum care. *Birth* 2001;28(4):254-63. doi: doi:
9. Rheindorf J, Hagist C, Schlereth C. Call (and pay) the midwife: A discrete choice experiment on mothers' preferences and their willingness to pay for midwifery care. *International Journal of Nursing Studies* 2021;124:104096. doi: doi:
10. Scotland GS, McNamee P, Cheyne H, et al. Women's preferences for aspects of labor management: results from a discrete choice experiment. *Birth* 2011;38(1):36-46. doi: doi:
11. Adams KP, Lybbert TJ, Vosti SA, et al. Using an economic experiment to estimate willingness-to-pay for a new maternal nutrient supplement in Ghana. *Agricultural Economics* 2016;47(5):581-95. doi: doi:
12. Brown LE. Applying stated-preference methods to health systems problems in sub-Saharan Africa. Johns Hopkins University, 2018.
13. Hanson K, McPake B, Nakamba P, et al. Preferences for hospital quality in Zambia: Results from a discrete choice experiment. *Health Economics* 2005;14(7):687-701. doi: doi:10.1002/hec.959
14. Morhason-Bello IO, Olayemi O, Ojengbede OA, et al. Attitude and preferences of Nigerian antenatal women to social support during labour. *Journal of Biosocial Science* 2008;40(4):553-62. doi: doi:10.1017/s0021932007002520
15. Nargesi DA, Hajizadeh M, Pakdel MJ, et al. Preferences of Iranians to select the emergency department physician at the time of service delivery. *BMC health services research* 2021;21:1155. doi: doi:https://dx.doi.org/10.1186/s12913-021-07183-9
16. Ozdemir S, Chen T, Tan CW, et al. Parturients' Stated Preferences for Labor Analgesia: A Discrete Choice Experiment. *Patient preference and adherence* 2022:983-94. doi: doi:

17. Umar N, Marchant T. What women want during institutional delivery: Exploring the dis-utilities associated with attributes of disrespect and abuse during institutional birth. *American Journal of Tropical Medicine and Hygiene* 2018;99:479-80. doi: doi:
18. Alcock G, Das S, More NS, et al. Examining inequalities in uptake of maternal health care and choice of provider in underserved urban areas of Mumbai, India: a mixed methods study. *BMC Pregnancy & Childbirth* 2015;15(1):1-11. doi: doi:10.1186/s12884-015-0661-6
19. Aremu O, Lawoko S, Dalal K. Neighborhood socioeconomic disadvantage, individual wealth status and patterns of delivery care utilization in Nigeria: A multilevel discrete choice analysis. *International Journal of Women's Health* 2011;3:167-74. doi: doi:https://dx.doi.org/10.2147/ijwh.s21783
20. Aziz RA, Zhuang J. A multi-attribute utility framework for patients to determine childbirth method considering uncertainties, patient preferences, risk attitudes, and pregnancy complications. *IISE Transactions on Healthcare Systems Engineering* 2022 doi: doi:https://dx.doi.org/10.1080/24725579.2022.2149637
21. Bharadwaj JS, Ganeshkumar P, Pattabi K. Determinants of Place of childbirth in Chamba District, Himachal Pradesh, India. *BMC Proceedings Conference: 1st Field Epidemiology Training Programs India Conference, FETP ICON* 2020;15 doi: doi:https://dx.doi.org/10.1186/s12919-021-00223-6
22. Dehury B, Chourase M. Does the preference for location of childbirth change for successive births? Evidence from the states and regions of India. *Journal of Biosocial Science* 2021;53(2):266-89. doi: doi:10.1017/S0021932020000188
23. Deleault JD. Treatment Decision Making in the Postpartum Period: Examining Women's Preferences and Perspectives. 2015 doi: doi:
24. Edmonds JK, Paul M, Sibley L. Determinants of place of birth decisions in uncomplicated childbirth in Bangladesh: An empirical study. *Midwifery* 2012;28(5):554-60. doi: doi:10.1016/j.midw.2011.12.004
25. Enuku CA, Makata NE, Elue O. Factors influencing preference for traditional birth attendant services compared to hospital care among reproductive age women in ugbekun community Benin City, edo state, Nigeria. *Annals of Biomedical Sciences* 2022;20(2):14-24. doi: doi:
26. Ibrahim R, Khalil R, Ehab R, et al. Preference of mode of delivery among women in childbear-ing period in egypt and factors affecting it. *Ginekologia i Poloznictwo* 2021;16 doi: doi:
27. Kanya L, Obare F, Bellows B, et al. Estimating willingness to pay for maternal health services: the Kenya reproductive health voucher programme. *African Health Monitor* 2015;20:43-50. doi: doi:
28. Konde A, Dolarno BL, Monareng LV. UGANDAN WOMEN'S CHILDBIRTH PREFERENCES. *Africa Journal of Nursing & Midwifery* 2011;13(2):3-13. doi: doi:
29. Kosan Z, Kavuncuoglu D, Calikoglu EO, et al. Delivery preferences of pregnant women: Do not underestimate the effect of friends and relatives. *Journal of Gynecology Obstetrics and Human Reproduction* 2019;48(6):395-400. doi: doi:https://dx.doi.org/10.1016/j.jogoh.2019.03.009
30. Mahdi SS, Habib OS. A study on preference and practices of women regarding place of delivery. *Eastern Mediterranean Health Journal* 2010;16(8):874-78. doi: doi:10.26719/2010.16.8.874
31. Mgudlwa B, Mavundla TR, Mbengo F, et al. SELF-REPORTED PREFERENCE FOR DELIVERY PLACE AMONG WOMEN PRESENTING FOR MATERNAL CARE HEALTH SERVICES AT A TERTIARY HOSPITAL IN THE EASTERN CAPE PROVINCE, SOUTH AFRICA. *Africa Journal of Nursing & Midwifery* 2017;19(1):157-69. doi: doi:10.25159/2520-5293/2109

32. Arije O, Madan J, Hlungwani T. Attributes development for a discrete choice experiment on preferences in sexual and reproductive health services for adolescents and young people in Nigeria. *BMC Health Services Research* 2022;22(1) doi: doi:10.1186/s12913-022-08888-1
33. Atukunda EC, Mugyenyi GR, Obua C, et al. Women's Choice to Deliver at Home: Understanding the Psychosocial and Cultural Factors Influencing Birthing Choices for Unskilled Home Delivery among Women in Southwestern Uganda. *Journal of pregnancy* 2020;2020:6596394. doi: doi:https://dx.doi.org/10.1155/2020/6596394
34. Chiwire P, Evers SM, Mahomed H, et al. Identification and Prioritization of Attributes for a Discrete Choice Experiment Using the Nominal Group Technique: Patients' Choice of Public Health Facilities in Cape Town, South Africa. *Value in Health Regional Issues* 2022;27:90-98. doi: doi:10.1016/j.vhri.2021.06.005
35. Jacobs C, Michelo C, Hyder A. Understanding maternal choices and experiences of care by skilled providers: voices of mothers who delivered at home in selected communities of Lusaka city, Zambia. *Frontiers in Global Women's Health* 2023;4 doi: doi:
36. Pokhrel S. Scaling up health interventions in resource-poor countries: What role does research in stated-preference framework play? *Health Research Policy and Systems* 2006;4 doi: doi:https://dx.doi.org/10.1186/1478-4505-4-4
37. Kachwaha S, Rao K, Kaplan A, et al. Women's Stated Preferences for Conditional Cash Transfer Programs Focused on Maternal and Child Health and Nutrition in India (P22-012-19). *Current Developments in Nutrition* 2019;3:nzz042. P22-12-19. doi: doi:
38. Ternent L. Using Discrete Choice Experiments to Elicit Preferences for Maternal Health Care in Ghana2007.
39. Beam NK, Dadi GB, Rankin SH, et al. A discrete choice experiment to determine facility-based delivery services desired by women and men in rural Ethiopia. *BMJ open* 2018;8(4):e016853.
40. Camacho EM, Smyth R, Danna VA, et al. Women's preferences for antenatal care in Tanzania: a discrete choice experiment. *BMC Pregnancy & Childbirth* 2022;22(1):1-10. doi: doi:10.1186/s12884-022-04634-x
41. Kruk ME, Paczkowski M, Tegegn A, et al. Women's preferences for obstetric care in rural Ethiopia: a population-based discrete choice experiment in a region with low rates of facility delivery. *Journal of Epidemiology & Community Health* 2010;64(11):984-88. doi: doi:10.1136/jech.2009.087973
42. Kruk ME, Paczkowski M, Mbaruku G, et al. Women's preferences for place of delivery in rural Tanzania: a population-based discrete choice experiment. *American Journal of Public Health* 2009;99(9):1666-72. doi: doi:10.2105/AJPH.2008.146209
43. Kumar M, Tele A, Kathono J, et al. Understanding depression treatment and perinatal service preferences of Kenyan pregnant adolescents: A discrete choice experiment. *PLoS ONE* 2023;18 doi: doi:https://dx.doi.org/10.1371/journal.pone.0273274
44. Larson E, Vail D, Mbaruku GM, et al. Moving toward patient-centered care in Africa: A discrete choice experiment of preferences for delivery care among 3,003 Tanzanian women. *PLoS ONE* 2015;10 doi: doi:https://dx.doi.org/10.1371/journal.pone.0135621
45. Mahumud RA, Alamgir NI, Hossain MT, et al. Women's preferences for maternal healthcare services in bangladesh: Evidence from a discrete choice experiment. *Journal of Clinical Medicine* 2019;8 doi: doi:https://dx.doi.org/10.3390/jcm8020132
46. Mazzoni A, Althabe F, Gutierrez L, et al. Women's preferences and mode of delivery in public and private hospitals: a prospective cohort study. *BMC Pregnancy & Childbirth* 2016;16:1-8. doi: doi:10.1186/s12884-016-0824-0

47. Oluoch-Aridi J, Adam MB, Wafula F, et al. Eliciting women's preferences for place of child birth at a peri-urban setting in Nairobi, Kenya: A discrete choice experiment. *PLoS ONE* 2020;15 doi: doi:https://dx.doi.org/10.1371/journal.pone.0242149
48. Oluoch-Aridi J, Adam MB, Wafula F, et al. Understanding what women want: Eliciting preference for delivery health facility in a rural subcounty in Kenya, a discrete choice experiment. *BMJ Open* 2020;10 doi: doi:https://dx.doi.org/10.1136/bmjopen-2020-038865
49. Paczkowski MM, Kruk ME, Tessema F, et al. Depressive symptoms and posttraumatic stress disorder as determinants of preference weights for attributes of obstetric care among Ethiopian women. 2012
50. Rajasulochana S, Nyarko E, Dash U, et al. Expectant Mother’s Preferences for Services in Public Hospitals of Tamil Nadu, India. *Journal of Health Management* 2016;18(2):305-17. doi: doi:10.1177/0972063416637745
51. Rijsbergen Bv, D'Exelle B. Delivery care in Tanzania: a comparative analysis of use and preferences. *World Development* 2013;43:276-87. doi: doi:
52. Umar N, Quaife M, Exley J, et al. Toward improving respectful maternity care: a discrete choice experiment with rural women in northeast Nigeria. *BMJ Global Health* 2020;5(3) doi: doi:
53. Wu L, Wu Y, Zou S, et al. Eliciting women's preference for prenatal testing in China: A discrete choice experiment. *BMC Pregnancy and Childbirth* 2020;20 doi: doi:https://dx.doi.org/10.1186/s12884-020-03270-7
